# From a Biosynthetic Pathway toward a Biocatalytic Process and Chemocatalytic Modifications: Three‐Step Enzymatic Cascade to the Plant Metabolite *cis*‐(+)‐12‐OPDA and Metathesis‐Derived Products

**DOI:** 10.1002/advs.201902973

**Published:** 2020-05-29

**Authors:** Jana Löwe, Karl‐Josef Dietz, Harald Gröger

**Affiliations:** ^1^ Chair of Industrial Organic Chemistry and Biotechnology Faculty of Chemistry Bielefeld University Universitätsstr. 25 33615 Bielefeld Germany; ^2^ Chair of Plant Biochemistry and Physiology Faculty of Biology Bielefeld University Universitätsstr. 25 33615 Bielefeld Germany

**Keywords:** biocatalysis, *cis*‐(+)‐12‐oxophytodienoic acid, metatheses, oxidations, oxylipins

## Abstract

A biotechnological approach toward the plant metabolite and regulator *cis*‐(+)‐12‐oxophytodienoic acid (*cis*‐(+)‐12‐OPDA) in a one‐pot process with >99% conversion, at least 90% selectivity and ≤10% of side products as well as a high diastereoselectivity (leading to d.r. of at least 90:10) is reported. The optimized organic‐synthetic enzyme cascade for preparing this bioactive and commercial molecule with pharmaceutical relevance on a gram per L scale is designed based on its biosynthetic pathway starting from cheap and readily accessible linolenic acid. Toward this end, a recombinant biocatalyst system has been prepared for carrying out the most critical two key steps in a tailored manner, thus avoiding sensitive intermediate decomposition. Furthermore, *cis*‐(+)‐12‐OPDA is successfully modified via a cross‐alkene metathesis reaction with conversions of up to >99%, leading to a compound library of new *cis*‐(+)‐12‐OPDA derivatives with different substitution pattern of the side chain at the 2‐position. By means of such a combined biotechnological and chemocatalytic route, a straightforward approach to a structurally unique oxylipin library is realized, which would be highly difficult or not accessible by pure chemical and biotechnological methods, respectively.

## Graphical abstract

A biocatalytic one‐pot cascade reaction toward the plant metabolite *cis*‐(+)‐12‐oxophytodienoic acid (*cis*‐(+)‐12‐OPDA) is developed, which enables its synthesis on a gram per L scale with excellent conversion of >99% and high selectivity of at least 90%. The product *cis*‐(+)‐12‐OPDA is subsequently successfully modified via a cross‐alkene metathesis reaction with conversions of up to >99%, leading to a compound library of new *cis*‐(+)‐12‐OPDA derivatives with diverse substitution pattern.

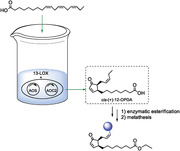

## 1 Introduction

Oxylipins are highly important signal molecules during stress regulation in plants being synthesized in cells by means of oxygenation of poly‐unsaturated lipids.^[^ ^1^ ^]^ Among such biologically relevant oxylipins are representatives of the product class of jasmonates. Jasmonates, like jasmonic acid (JA), methyl jasmonate (MeJA) and derivatives^[^ ^2^ ^]^ are responsible for defense reactions after wounding or pathogen infestation. Furthermore, they are essential in the reproduction of plants.^[^ ^3^ ^]^

**Figure 1:**
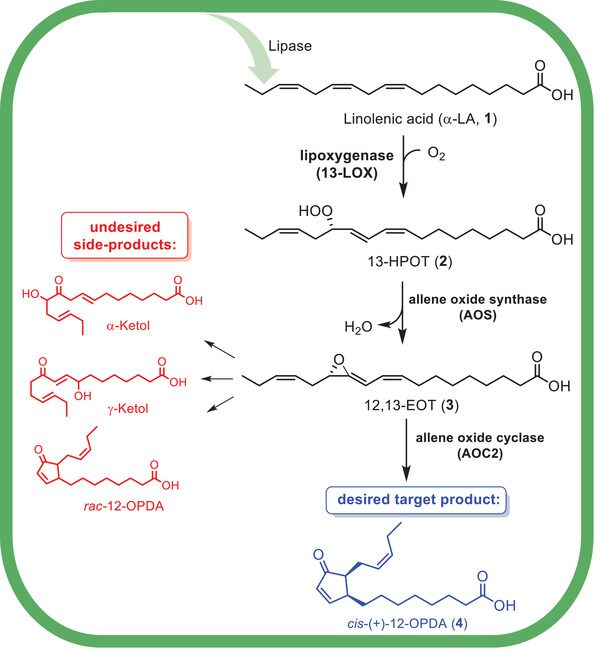
Biosynthetic pathway toward *cis*‐(+)‐12‐OPDA (**4**) and undesired side products.

The biosynthesis of jasmonic acid in planta starts with the liberation of α‐linolenic acid (**1**) by means of acylhydrolases or phospholipases out of the chloroplast membrane (**Figure** **1**).^[^ ^4^ ^]^ The α‐linolenic acid (**1**, α‐LA) is then converted in the presence of a 13‐lipoxygenase (13‐LOX), which catalyzes the peroxidation of this unsaturated fatty acid by insertion of oxygen at position 13 in the aliphatic unsaturated chain of α‐LA (**1**). The resulting (*S* )‐13‐hydroperoxy‐octadecatrienoic acid (**2**, 13‐HPOT) is then processed by an allene oxide synthase (AOS). AOS‐enzymes originate from the cytochrome CYP450 oxidoreductase superfamily; they are subordinated to the subfamily of CYP74.^[^ ^1^ ^]^ These enzymes catalyze the rearrangement of the fatty acid peroxide (13‐HPOT, **2**) to the corresponding fatty acid epoxide, (9*Z*,13*S*,15*Z* )‐12,13‐epoxy‐octadecatrienoic acid (**3**, 12,13‐EOS). The highly unstable 12,13‐EOS (**3**) cyclizes without any enzyme to racemic 12‐oxo‐phytodienoic acid (*rac*‐12‐OPDA) or the α‐ or γ‐ketol (see Figure 1), whereas in the presence of an allene oxide cyclase the reaction is catalyzed to form *cis*‐(+)‐12‐oxo‐phytodienoic acid (*cis*‐(+)‐12‐OPDA, **4**).^[^ ^5^ ^]^ The reaction toward 12‐OPDA, **4**, was firstly described by Vick and Zimmerman in 1979.^[^ ^6^ ^]^ This biogenetic jasmonic acid precursor **4** shows a biological activity in plants as well.^[^ ^7^, ^8^ ^]^ 12‐OPDA (**4**) is then transported into the peroxisomes via ABC transporters or an ion trapping mechanism.^[^ ^9^ ^]^ Afterward, the double bond of the cyclopentenone ring is reduced by the ene‐reductase OPR3. This is followed by a triple β‐oxidation in which the aliphatic side chain of the 12‐OPDA (**4**) is shortened. This shortening of the chain results in the formation of (3*R*,7*S*)‐*cis*‐jasmonic acid, which rearranges to the more stable (3*R*,7*R*)‐*trans*‐jasmonic acid.^[^ ^10^ ^]^

In addition to the role in signal transduction in planta, jasmonic acid is a very important ingredient in the perfume industry underlined by a review from Chapuis about activites at Firmenich as a leading fragrance producer.^[^ ^11^ ^]^ Jasmonic acid and some derivatives such as, e.g., MeJA or the saturated Hedione are known for their exquisite jasmine fragrance and are used in many perfumes.

It is noteworthy that these molecules are accessible today in industry through very tedious multistep syntheses.^[^ ^11^ ^]^ The starting point of this synthesis is typically a cyclopentenone derivative followed by a multistep synthesis, whereas in nature the starting material is the unsaturated fatty acid **1** followed by a three‐step enzymatic synthesis. Due to the simple fatty acid substrate and short route of the biosynthetic pathway, we became interested to transform this knowledge of the natural synthesis of *cis*‐(+)‐12‐OPDA (**4**) in plant cells according to Figure 1 into an efficient organic‐synthetic process. This process should provide a synthetically useful access toward *cis*‐(+)‐12‐OPDA (which is presently only commercially available at a very high price, e.g., for 840 Euro per milligram)^[^ ^12^ ^]^ and on a long‐term, jasmonates. Furthermore, we were interested to build up compound libraries of *cis*‐(+)‐12‐OPDA derivatives for getting a deeper insight into structure‐activity relationships of this plant hormone and pharmaceutically as well as agriculturally relevant compound *cis*‐(+)‐12‐OPDA (**4**).

## 2 Results and Discussion

### 2.1 The Concept for Designing a Biocatalytic Cascade Process for *cis*‐(+)‐12‐OPDA

With respect to the first goal of developing an organic‐synthetic process in analogy to the biosynthetic pathway shown in Figure 1,^[^ ^13^ ^]^ in literature already some enzymatic syntheses of *cis*‐(+)‐12‐OPDA (**4**) on analytical scale are shown,^[^ ^14^, ^15^, ^16^, ^17^ ^]^ which, however, lack in process efficiency and suffer from low yields as well as tedious workup due to, e.g., side‐product formation. Targeting an efficient and scalable biocatalytic production of 12‐OPDA **4**, our aim was to combine all three reaction steps in a one‐pot cascade. The initial step for the successful synthesis of *cis*‐(+)‐12‐OPDA (**4**) consisted in getting an attractive access toward all three enzymes, the 13‐LOX, the AOS and AOC. Therefore, the commercially available 13‐LOX from *Glycine max* was chosen and the AOS and AOC2 were produced as recombinant enzymes from *Arabidopsis thaliana* (AtAOS and AtAOC2).^[^ ^17^ ^]^ Toward this end, two genes encoding for AtAOS and AtAOC2 were purchased from ThermoScientific as genes in pET28a for AtAOS with an N‐terminal peptide‐tag (AKKTSS^[^ ^18^ ^]^) and AtAOC2 without chloroplast target sequence. The concept is based on the coexpression of AtAOS and AtAOC2 and, thus, simultaneous use of the two enzymes AOS and AOC in one whole cell, because it is literature known that there is a proximity of both enzymes in terms of time and place.^[^ ^19^ ^]^ We envisaged that such a coexpression and the availability of both enzymes in one cell could be in particular beneficial in order to ensure a rapid conversion of the unstable allene oxide intermediate **3**, thus suppressing the formation of side products. An advantage of utilizing directly whole cells compared to isolated enzymes (used as crude extract) is that by means of whole cells one can make use of the total amount of the overexpressed AtAOS enzyme. In contrast, due to the high hydrophobicity of the membrane‐bound AtAOS^[^ ^20^ ^]^ their concentration in a crude extract is expected to be low as such enzymes remain attached to the membrane after centrifugation.

### 2.2 Process Design for the First Reaction Step: Lipoxygenase‐Catalyzed Peroxide Formation

As the organic peroxide 13‐HPOT (**2**) represents the first intermediate in the 12‐OPDA (**4**)‐synthesis, its enzymatic formation by means of 13‐LOX starting from α‐linolenic acid (**1**) as a substrate was investigated as well as the properties of the biocatalyst 13‐LOX in particular in terms of stability under process conditions as 13‐HPOT (**2**) is a relatively reactive and difficult‐to‐handle compound. Therefore, the effect of several parameters on the reaction was studied. The transformation of the fatty acid **1** toward 13‐HPOT (**2**) was monitored by means of a spectrophotometric assay (see Supporting Information, Section 3.2). After studying in detail the impact of the type of buffer, the concentration of the salt, pH‐value, temperature and amount of ethanol as a cosolvent, the best results were achieved with NH_4_Cl‐buffer, 100 × 10^−3^ m buffer concentration, pH 9, at 25–35 °C and with 5% ethanol (see Supporting Information), and we found that the activity decreases with lower pH (7–8) and with less amount of salt. Obviously, the higher salt amount has a positive impact toward the activity of 13‐LOX, presumably due to a stabilizing effect. The observed pH‐influence might be explained by a decreased ionic interaction of the positively charged arginine in the active site of the 13‐LOX and the carboxylate of α‐linolenic acid (**1**) at lower pH values due to a larger portion of the protonated form of the carboxylate.^[21]^ With this first set of optimized reaction parameters in hand, next the effect of further parameters having an impact on process and product stability was studied such as reaction time, reaction temperature and the way how to treat the mixture with oxygen (**Table** **1**). The reactions were performed in a practical and easy‐to‐conduct setup under preferably a continuous oxygen stream in an ammonium chloride buffer. The yield of the obtained 13‐HPOT (**2**) increased with oxygen saturation of the reaction system, thus indicating an oxygen limitation of this biotransformation (being aware that a quantification of this effect by means of measuring oxygen transfer rates still has to be done in future work). Thus, an oxygen saturation is beneficial for a high reaction rate, which has been achieved by the continuous oxygen stream. The oxygen saturation also enabled to reduce the required reaction time. A short reaction time is advantageous as less side products are formed (which was proven by ^1^H‐NMR spectroscopy), thus being beneficial for a formation of this unstable compound 13‐HPOT (**2**) in high yield. In addition, the reaction temperature plays an important role, and a higher enzyme activity occurred at an elevated temperature of 25 °C (and, thus a faster reaction rate of the 13‐LOX‐catalyzed peroxide formation) compared to a lower reaction temperature of 4 °C.

**Table 1:**
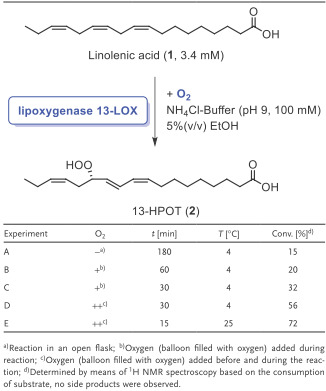
Lipoxygenase (13‐LOX)‐catalyzed reaction toward 13‐HPOT (**2**)

It should be added that also the linoleic acid‐derived esters methyl linoleate (**5**) and ethyl linoleate (**6**) were tested via a spectrophotometric assay as the use of such substrates could improve the permeability through the membrane of the whole cell catalyst and simplify the workup after the reaction. The activity was examined at several pH‐values (pH 7–9). However, the activity toward the esters turned out to be very poor at each pH‐value (Table S4, Supporting Information). This result underlines the hypothesis of the (above mentioned) strong impact of the interaction of the carboxylic residue of 13‐LOX and the positive charged arginine.

### 2.3 Whole Cell‐Catalyst Design for the Second and Third Reaction Steps and Proof‐of‐Concept for the Biocatalytic Three‐Step Cascade One‐Pot Process

**Figure 2:**
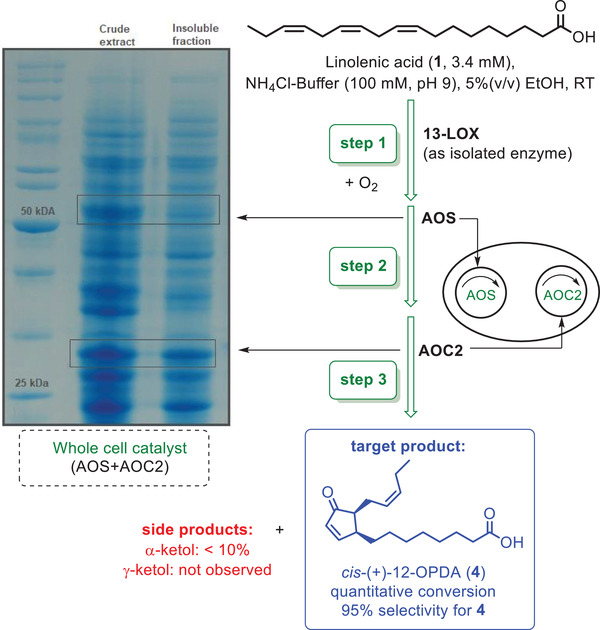
Reaction scheme for the synthesis of *cis*‐(+)‐12‐OPDA (**4**).

The knowledge of these reaction parameters then served as an ideal starting point for the design of the one‐pot synthesis of *cis*‐(+)‐12‐OPDA (**4**) starting from linolenic acid (**1**). The concept of this one‐pot synthesis is shown in **Figure** **2**. With the optimized transformation of linolenic acid to 13‐HPOT (**2**) as step 1 in hand, next a suitable whole cell catalyst for catalyzing steps 2 and 3 was designed in order to finally combine all steps. For this two‐step transformation of 13‐HPOT into 12‐OPDA a whole cell catalyst (WCC) consisting of AtAOS and AtAOC2 was prepared, in which the activities of both enzymes were adjusted in a favorable way, thus preventing the decomposition of the unstable allene oxide **3** as most labile intermediate within this cascade. In detail, we used genes encoding for AtAOS in pET28a(+) and AtAOC2 in pUC18 and pQE30, both without chloroplast target sequence. The activity assay for the AOS and AOC2 was representatively done with the AOS only as 13‐HPOT (**2**) can be isolated as a semistable substrate for activity assay in contrast to the unstable intermediate 12,13‐EOT (**3**). AtAOS displayed its best activity in NaPi‐buffer at 20 °C with 5% ethanol (see Supporting Information). In addition, both enzymes were tested with and without *Escherichia coli* codon optimization. The codon optimization led to a better overexpression but had a negative impact toward activity because of misfolding of the protein (indicated by the large amount of inclusion bodies in this case). The constructs without codon optimization were transformed into *E. coli* BL21(DE3) and *E. coli* BL21‐CodonPlus‐RIL and expressed, and the resulting whole cell catalysts were then used for the one‐pot synthesis.

With the readily available 13‐LOX (for step 1) and the tailor‐made whole cell catalyst *E. coli* BL21‐CodonPlus‐RIL containing AtAOS and AtAOC2 (for steps 2 and 3) in hand, then the desired practical and easy‐to‐conduct one‐pot synthesis was performed utilizing the commercially available 13‐LOX and the constructed tailor‐made whole cell catalyst, consisting of AtAOS and AtAOC2 in recombinant form (Figure 2). The reaction was performed at room temperature with oxygen saturation in a straightforward reaction setup. These reaction conditions were chosen because of the promising results for the synthesis of 13‐HPOT (**2**). An ammonium chloride buffer was used with a pH of 9 (based on the results of the activity assay) in combination with a small amount of ethanol to enhance the solubility of α‐linolenic acid (**1**) in the aqueous reaction medium.

When conducting the reaction with separate cells (pET28a‐AtAOS and pET28a‐AtAOC2) a full consumption of the substrate was observed. However, the amount of desired *cis*‐(+)‐12‐OPDA (4) was only 69% while 31% of side product (α‐ketol) was formed. The transformation to product **4** also works without AtAOC2 but led to only 23% of a 12‐OPDA product (which, however, is formed as a racemate in this case in accordance with literature^[^ ^15^ ^]^) and an increased amount of the α‐ketol side product of 77% whereas no γ‐ketol was observed. With AtAOC2 in pUC18, 69% product and 31% side product (α‐ketol) were formed. In contrast, when using AtAOC2 in pQE30 we were pleased to find that formation of side products is highly suppressed while achieving a high conversion toward *cis*‐(+)‐12‐OPDA (**4**): in detail, when conducting the one‐pot bioprocess under these conditions at a substrate loading of 1 g L^−1^ (being already in a good range for a metabolite synthesis) and at a 100 mg scale, the formation of the α‐ketol side product dropped to less than 10% and again the γ‐ketol was not formed at all. In addition, the substrate **1** was fully consumed and the conversion related to the formation of the desired *cis*‐(+)‐12‐OPDA (**4**) raised up to 95% in combination with a diastereomeric ratio of d.r.(*cis:trans*)=90:10. After (nonoptimized) workup, which suffered from product loss, a yield of 28% was obtained for **4** in this initial biotransformation experiment.

### 2.4 Biocatalytic Cascade Process at Elevated Lab Scale

In addition, a biotransformation at an elevated lab scale of ≈230 mL and at an increased substrate loading of 2 g L^−1^ linolenic acid (**1**) was successfully conducted. Starting from 456 mg (1.64 mmol) of substrate **1**, again full consumption of **1** was observed in combination with strong suppression of side‐product formation as well as a high selectivity of 91% for 12‐OPDA **4**. In the workup comprising extraction and subsequent automatic reverse phase column chromatography, the side product was easily separated and an amount of 150 mg of the purified target product **4**, corresponding to a yield of 31%, was obtained (d.r.(*cis:trans*)=90:10). To the best of our knowledge, this represents the first example of a successful combination of all three enzymes 13‐LOX, AOS and AOC2 in a one‐pot process in combination with a tailor‐made whole cell catalyst to obtain the biologically active product 12‐OPDA **4** with excellent conversion, high selectivity (of >90% for **4** and <10% of ketol side product) and high diastereomeric ratio (with d.r.(*cis:trans*) of at least 90:10) as well as in high purity and at a (relatively) high product concentration (considering that this compound **4** is a “classic biosynthetic metabolite”). The loss of product **4** in the downstream processing can be explained by the literature‐known challenge to isolate lipid‐type compounds from biomass and aqueous media due to the emulsifying properties of such products.^[^ ^22^, ^23^ ^]^ However, we were pleased to find that even in an initial optimization the yield could be nearly doubled to 61% (d.r.(*cis:trans*) =94:6) via a sonification strategy in the extraction step (see Supporting Information), which we attribute to an improved extraction of a large portion of 12‐OPDA **4**, which remained in the cell pellet due to hydrophobic interactions with the cell membrane. In addition, the optical rotation of this purified *cis*‐(+)‐12‐OPDA (**4**) sample (d.r.(*cis/trans*) =94:6) has been measured and indicated a high enantiomeric purity by comparison with the literature data^[^ ^24^ ^]^ for enantiomerically pure *cis*‐(+)‐12‐OPDA (**4**).

Being aware that still the product isolation and resulting yields need to be optimized (as a task being currently under investigation but being beyond the scope of this proof of concept for a highly selective cascade), at the same time it should be stated that by “translating” this biosynthetic pathway into a synthetically applicable bioprocess such a complex molecule as **4** can now be prepared within a one‐pot process starting from the simple and cheap fatty acid (**1**) in comparison to many synthetic steps with tedious workup operations in the organic synthetic routes,^[^ ^8^, ^11^ ^]^ which additionally start from more complex substrates.

### 2.5 Merging the Bioprocess for 12‐OPDA with Metal‐Catalyzed Metathesis and Construction of a Compound Library of 12‐OPDA Derivatives for Biological Studies

With this efficient process for *cis*‐(+)‐12‐OPDA (**4**) in hand (which now enables a convenient synthesis of this compound **4** in gram amount), as a next step we focused on a strategy for a straightforward preparation of a library of 12‐OPDA derivatives showing a different substituent pattern in the α‐position. For such a desired chemical modification of 12‐OPDA (**4**) we envisaged to utilize the metal‐catalyzed alkene‐cross metathesis (CM) as an attractive method applied in various natural product syntheses.^[^ ^25^ ^]^ Its efficient formation of carbon–carbon double bonds^[^ ^26^ ^]^ with robust ruthenium catalysts makes this method an important synthetic tool for the production of a range of biologically active organic molecules.^[^ ^27^ ^]^ Moreover CM runs at mild reaction conditions and is therefore used in several total syntheses of natural products.^[^ ^28^ ^]^ A selected example is the recently reported total synthesis of Δ12‐prostaglandin,^[^ ^29^ ^]^ which can be regarded as a *cis*‐(+)‐12‐OPDA (**4**)‐related metabolite.^[^ ^8^ ^]^ Also the synthesis of jasmonic acid derivatives was accomplished via olefin‐cross metathesis.^[^ ^30^ ^]^

Due to these advantages, we chose the CM method in order to modify *cis*‐(+)‐12‐OPDA (**4**) and generate a new, diverse library of *cis*‐(+)‐12‐OPDA (**4**)‐derived potentially bioactive molecules (**Table** **2**). In particular, in continuation of our previous studies on *cis*‐(+)‐12‐OPDA derivatives,^[^ ^17^ ^]^ we became interested in modifying the length as well as substitution pattern of the side chain at the C2‐position of 12‐OPDA **4**. For the metathesis reactions, we chose the Hoveyda–Grubbs 2nd‐generation catalyst (**C1**) based on its successful applications in literature syntheses and its readily commercial availability.^[^ ^25^, ^26^, ^27^, ^28^, ^29^, ^30^, ^31^, ^32^ ^]^ As a solvent for this initial lab scale metathesis‐type synthesis we used methylene chloride which turned out as a preferred solvent for metathesis catalysis.^[^ ^33^ ^]^

**Table 2:**
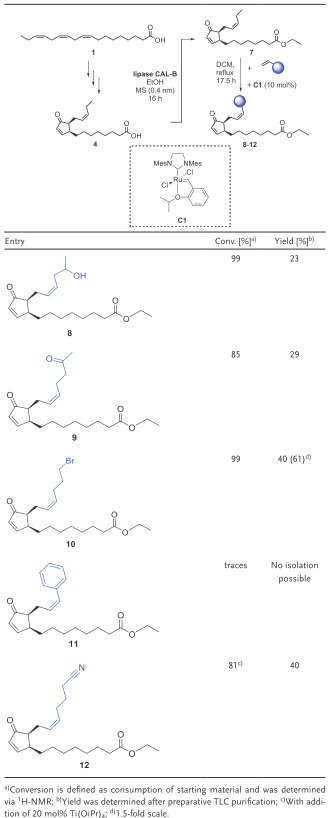
Construction of a library of *cis*‐(+)‐12‐OPDA (**4**)‐derived molecules based on lipase‐catalyzed esterification and subsequent metathesis reactions

Starting from *cis*‐(+)‐12‐OPDA (**4**) being synthesized by means of the three‐step enzyme cascade as described above, an enzymatic esterification was carried out utilizing lipase B from *Candida antarctica* (CAL‐B). Such an enzymatic esterification has the advantage of mild reaction conditions, and therefore less isomerization should occur. This hypothesis has been proven by the experimental results. When conducting the esterification of the *cis*‐configured **4** with lipase B from *C. antarctica*, a high *cis*:*trans*‐ratio of 90:10 was obtained for the resulting ester whereas when applying chemical esterification protocols under more harsh conditions full isomerization to the *trans*‐isomer was observed. These findings are of general interest as it will enable the study of *cis*‐ and *trans*‐esters of **4** in biological tests in the future.

Subsequently, alkene‐cross metathesis reactions were done with the *cis*‐(+)‐12‐OPDA‐derived ester **7** and different types of olefins in the presence of catalyst **C1** (Table 2). In most cases, high to excellent conversions were achieved, which is underlined by the successful formation of the metathesis products **8**–**10** and **12** with conversions in the range of 81–99%. An exception leading to low conversion, however, is the metathesis reaction with styrene as reagent. In addition, in case of the metathesis reaction of *cis*‐(+)‐12‐OPDA ester **7** with hex‐5‐enennitrile, the addition of 20 mol% Ti(OiPr)_4_^[^ ^31^ ^]^ was necessary for full consumption of substrate **7**. It is noteworthy that no self‐metathesis product of *cis*‐(+)‐12‐OPDA‐derived ester **7** was observed, thus leading to the formation of the products with excellent selectivity.

As the desired metathesis reactions proceeded smoothly, a range of new *cis*‐(+)‐12‐OPDA (**4**)‐derived molecules (**8**–**10**, **12**) were obtained, which now can be used as a first small compound library of *cis*‐(+)‐12‐OPDA‐derivatives for biological tests. It should be noted that yields in the metathesis reactions so far have not been optimized as our major focus was on the construction of such a compound library for biological studies. Process development and optimization of the metathesis products will be done after identifying a suitable hit in these biological studies, which is then needed in larger amount. At present the conversions are already very good to excellent (with 81–99%) with exception of the metathesis product derived from styrene. After isolation, yields so far are in the range of up to 61% which is due to a nonoptimized workup protocol and the small scale of these reactions (mg scale). The potential for higher yields at an elevated lab scale is underlined by an already achieved increase of the yield from 40% to 61% when just using a 1.5‐fold larger scale of the reaction for the preparation of the metathesis product **10** (Table 2).

## 3 Conclusion

In conclusion, inspired from a biosynthetic pathway we developed an efficient biocatalytic one‐pot process for *cis*‐(+)‐12‐OPDA (**4**) as a synthetically challenging plant metabolite product being of pharmaceutical and agricultural relevance, which until now could only be synthesized in small mg amount, low yield and based on a tedious workup. In contrast, the tailor‐made design of a one‐pot process based on the use of a lipoxygenase and a constructed whole‐cell catalyst carrying further two of the three essential enzymes for this transformation led to an efficient synthesis of *cis*‐(+)‐12‐OPDA (**4**) with full substrate consumption, high diastereoselectivity (leading to diastereomeric ratio of at least 90:10), low side‐product formation (not exceeding 10%) and high selectivity (of at least 90%) with respect to product formation in spite of the presence of labile intermediates within this cascade. Further advantages are the use of linolenic acid (**1**) as a readily available, cheap substrate as well as a simple workup step and the access to a purified *cis*‐(+)‐12‐OPDA (**4**) product in good yield. These results could be also of interest for further plant physiological studies as this route represents an attractive route to *cis*‐(+)‐12‐OPDA (**4**), which gains increasing attention in this field.^[^ ^34^ ^]^ Due to the availability of *cis*‐(+)‐12‐OPDA (**4**) in larger amount by means of this new bioprocess, we also prepared a compound library of *cis*‐(+)‐12‐OPDA derivatives, which were accessible through alkene‐cross metathesis. Such libraries are of interest for getting insight into structure‐bioactivity relationships as well as for identifying new pharmaceutically interesting target compounds based on *cis*‐(+)‐12‐OPDA (**4**).

## 4 Experimental Section

##### Materials

The compound α‐linolenic acid (**1**) as raw material for the biotransformation, the lipoxygenase from *Glycine max* (13‐LOX; Type I‐B, lyophilized powder, Sigma Aldrich‐L7395, 50.000 U mg^−1^ solid), the Hoveyda–Grubbs catalyst 2nd generation (**C1**) and reagents for the metathesis reactions as well as the organic solvents used in this study were purchased commercially and used as received.

##### Enzyme Sequence Data, Biocatalyst Preparation, and Characterization

The gene and protein sequences of the utilized allene oxide cyclase (AOC2) from *Arabidopsis thaliana* and AOS from *Arabidopsis thaliana*, respectively, and their plasmid maps as well microbiological procedures for the preparation of the *E. coli* strains and biochemical characterization of the enzymes (including expression data such as SDS‐PAGE as well as data related to enzyme activities) are given in the Supporting Information.

##### Procedure for the Biocatalytic Synthesis of 13‐HPOT (**2**)

The starting material α‐linolenic acid (**1**) was diluted in ethanol (1 mL), and the resulting solution was dissolved in an ammonium chloride buffer (100 × 10^−3^ m, pH 9). To this solution, the lipoxygenase from *Glycine max* (40 U), which was also dissolved in ammonium chloride buffer (100 × 10^−3^ m, pH 9), was added. The reaction was performed under oxygen saturation at room temperature, and the reaction was monitored via TLC (cyclohexane/EtOAc/AcOH 6:2:0.1 v/v). Afterward the reaction mixture was acidified to pH 2 with hydrochloric acid (2 m) and extracted twice with DCM (1:1, v/v). Magnesium sulfate was added to remove residual water. The solvent was removed via rotary evaporator and the composition of the reaction mixture was analytically determined. For more details, see Table 1 as well as the Supporting Information.

^1^H NMR (500 MHz, CDCl_3_) δ (ppm): 6.58 (ddt, *J* = 15.3, 11.1, 1.0 Hz, 1H), 5.99 (t, ^3^ *J* = 11.0 Hz, 1H;) 5.58 (dd, ^3^ *J* = 8.0 Hz ^4^ *J* = 15.2, 1H), 5.53 – 5.47 (m, 2H), 5.33 (dtt, ^3^ *J* = 7.3 Hz, ^4^ *J* = 10.7, 1.7 Hz, 1H), 4.43 (dt, ^3^ *J* = 8.2, 6.6 Hz, 1H), 2.50 – 2.44 (m, 1H), 2.34 (t, ^3^ *J* = 7.4 Hz, 2H), 2.32 – 2.28 (m, 1H), 2.18 (dtd, ^3^ *J* = 7.4 Hz, ^4^ *J* = 1.5, 15.0 Hz, 2H), 2.05 (pd, ^3^ *J* = 7.4 Hz, ^4^ *J* = 1.5 Hz, 2H), 1.63 (p, ^3^ *J* = 7.3 Hz, 3H), 1.25 (td, ^3^ *J* = 7.1, 4.6 Hz), 0.96 (t, ^3^ *J* = 7.5 Hz, 3H).

^13^C NMR (126 MHz, CDCl_3_) δ (ppm): 178.46, 134.53, 134.26, 130.42, 130.36, 127.68, 123.22, 86.36, 33.89, 30.76, 29.42, 29.00, 28.94, 27.80, 24.77, 20.85, 14.25.

EI‐MS [*m*/*z*]: 333.26 [M+Na]^+^349.17 [M+K]^+^.

HRMS (ESI): calculated for [M+Na]^+^ 333.2036, found 333.2043.

FT‐IR [cm^−1^]: 3500–3000 (OH‐ ν), 2924 (CH‐ ν), 2853 (CH‐ ν), 1704 (C = O‐ ν), 1456–1409 (CH_3_/CH_2_‐δ), 1243 (CH_3_ ‐δ), 1200 ((*E*)‐CH_2_‐progression bands), 948 (OH‐δ).

The ^1^H and ^13^C NMR spectra of isolated 13‐HPOT (**2**) are shown in the Supporting Information.

##### Procedure for the Biocatalytic Three‐Step One‐Pot Cascade Process for *cis*‐(+)‐12‐OPDA (**4**)

An ammonium chloride buffer (100 mL, 100 × 10^−3^ m, pH 9) was saturated with oxygen, and *E. coli* BL21(DE3)CodonPlusRIL cells containing AtAOS and AtAOC2 (300 mg) or AtAOS (150 mg) and AtAOC (150 mg) were suspended in the same buffer (ammonium chloride buffer, 100 × 10^−3^ m, pH 9). The resulting mixture was again saturated with oxygen for five minutes. Subsequently, lipoxygenase from *Glycine max* (3 mg, 40 U, dissolved in ammonium chloride buffer (1 mL, 100 × 10^−3^ m, pH 9)) was added to this mixture, which then was once more saturated with oxygen for five minutes. Then, the reaction was started by addition of α‐linolenic acid (**1**, 100 mg, 0.36 mmol dissolved in ethanol (5 mL)). The reaction was performed under oxygen saturation at room temperature, and the progress of the reaction was monitored via TLC (cyclohexane/EtOAc/AcOH 6:2:0.1 v/v). After a reaction time of 1 h, the reaction mixture was centrifuged (10 000 × *g*, 15 min, 4 °C). The supernatant was acidified with 2 m hydrochloric acid (4 mL) and extracted with DCM (3 × 50 mL). Magnesium sulfate was added to remove residual water. The solvent was removed in vacuo, and subsequently the crude product was purified via automated column chromatography on a C18‐column (MeCN/H_2_O/AcOH; ACN/H_2_O, gradient 0–100% ACN and AcOH (0.1% v/v)). The desired product *cis*‐(+)‐12‐OPDA (**4**) was isolated and the purity of the product was determined via ^1^H NMR spectroscopic analysis. For more details, see the Supporting Information.

Yield: 27.8 mg (0.10 mmol), 28%.

^1^H NMR (500 MHz, CDCl_3_): δ (ppm) = 7.73 (dd, ^3^ *J* = 6.0, ^4^ *J* = 2.7 Hz, 1H), 7.60 (d, ^3^ *J* = 5.7 Hz, 1H), 6.18 (dd, ^3^ *J* = 5.9, ^4^ *J* = 1.7 Hz, 1H), 6.12 (d, ^3^ *J* = 5.9 Hz, 1H)_,_ 5.46 – 5.32 (m, 2H), 2.97 (ddt, ^3^ *J* = 5.9, 10.8, 7.6, ^4^ *J* = 3.6 Hz, 1H), 2.50 (dt, ^3^ *J* = 15.3, 5.4 Hz, 1H), 2.47 – 2.41 (m, 1H), 2.35 (t, ^3^ *J* = 7.5 Hz, 2H), 2.17 – 2.10 (m, 1H), 2.06 (d, ^3^ *J* = 7.5 Hz, 2H), 1.72 (td, ^3^ *J* = 11.1, 5.0 Hz, 1H), 1.63 (p, ^3^ *J* = 7.2 Hz, 3H), 1.32 (q, ^3^ *J* = 7.1, 5.9 Hz, 8H), 1.15 (dtd, *J* = 14.3, 9.6, ^4^ *J* = 4.5 Hz, 1H), 0.97 (t, ^3^ *J* = 7.5 Hz, 3H).

The spectral data are according to literature.^[^ ^17^ ^]^ The ^1^H NMR spectrum of isolated *cis*‐(+)‐12‐OPDA (**4**) is shown in the Supporting Information.

##### Procedure for the Metathesis Reactions Exemplified for the Metathesis Product ***10***

A solution of the ethyl ester of *cis*‐(+)‐12‐OPDA (**7**, 30.0 mg, 0.09 mmol) and 5‐bromopentene (41.0 µL, 0.34 mmol, 4 equiv.) in degassed dichloromethane (10 mL) was treated with the Hoveyda–Grubbs catalyst 2nd generation (**C1**, 13.3 mg, 0.016 mmol, 18 mol%), and the resulting reaction mixture was stirred for 17.5 h. Afterward the solvent was removed in vacuo, and the resulting crude product was purified via thin layer chromatography (eluent: cyclohexane/EtOAc v/v 3:1) to furnish the desired metathesis product **10** in purified form.

Yield: 23 mg (56 µmol), 61%.

^1^H NMR (500 MHz, CDCl_3_): δ (ppm) = 7.72 (dd, ^3^ *J* = 5.8, ^4^ *J* = 2.7 Hz, 1H), 6.16 (d, ^3^ *J* = 5.8 Hz, 1H), 5.61 – 5.36 (m, 2H), 4.11 (q, ^3^ *J* = 7.2 Hz, 2H), 3.38 (dt, ^4^ *J* = 15.1, ^3^ *J* = 6.6 Hz, 2H), 3.01 – 2.94 (m, 1H), 2.60 (m, 2H), 2.50 (m, 1H), 2.47 – 2.36 (m, 1H), 2.28 (t, ^3^ *J* = 7.5 Hz, 2H), 2.20 –2.15 (m, 1H), 1.90 (m, 2H), 1.77 – 1.68 (m, 1H), 1.67 – 1.58 (m, 2H), 1.31 (s, 8H),1.25 (t, ^3^ *J* = 7.1 Hz, 4H).

^13^C NMR (126 MHz, CDCl_3_): δ (ppm) = 210.73, 173.96, 167.18, 132.61, 130.18, 129.59, 60.34, 49.43, 44.46, 34.47, 33.34, 32.42, 31.04, 30.97, 29.75, 29.30, 29.21, 28.88, 27.73, 25.05, 14.42.

MS (ESI): *m*/*z* = 435.229 *m*/*z* [M+Na]^+^, 437.186 *m*/*z* [M+Na]^+^.

HRMS (ESI): calculated for [M+Na]^+^ 435.1505, found 435.1506.

IR (neat) [cm^−1^]: 2925 (s, ν, CH_2_), 2854 (m, ν, CH_2_), 1732 (s, ν, C=O‐Ester), 1702 (vs, ν, C=O‐carbonyl), 1586 (w, ν, C=C), 600 (w, ν, C‐Br).

The ^1^H and ^13^C NMR spectra of isolated metathesis product **10** are shown in the Supporting Information.

## Conflict of Interest

The authors declare no conflict of interest.

## Supporting information

Supporting Information
