# Supplementary material for: From a Biosynthetic Pathway toward a Biocatalytic Process and Chemocatalytic Modifications: Three‐Step Enzymatic Cascade to the Plant Metabolite cis‐(+)‐12‐OPDA and Metathesis‐Derived Products
Source: Adv Sci (Weinh). 2020 May 29;7(13):1902973. doi: 10.1002/advs.201902973 (PMC7341106; doi:10.1002/advs.201902973)
Supplement: Supplementary file 1 — Supporting Information [file ADVS-7-1902973-s001.pdf]

WILEY-VCH

Copyright WILEY-VCH Verlag GmbH & Co. KGaA, 69469 Weinheim, Germany, 2018.

Supporting Information

From a biosynthetic pathway toward a biocatalytic process and chemocatalytic modifications: Three-step enzymatic cascade to the plant metabolite *cis*-(+)-12-OPDA and metathesis-derived products

*Jana Löwe, Karl-Josef Dietz, Harald Gröger**

Table of content

Experimental Information.................... 4
- Sequences and plasmid maps.................... 4
- pET28a-AtAOC2 _WTΔchlp(C)His.................... 4
- pQE30-AtAOC2 _WTΔchlp(N)His.................... 5
- pUC18-AtAOC2 _WTΔchlp(N)His.................... 7
- pET28a-AtAOS _WTΔchlp(C)His.................... 8
- Microbiological and biochemical procedures....................10
- Production of competent cells....................10
- Transformation of competent cells with plasmid-DNA....................10
- Production of whole cell catalyst....................10
- Heterologous expression of AtAOS and AtAOC....................10
- Cell Disruption....................11
- Biochemical methods....................11
- SDS-PAGE Analysis....................11
- Activity Assay....................16
- Activity assay 13-LOX....................18
- Activity assay 13-LOX and derivatives at different pH....................19
- Activity assay AtAOS....................20
- Chemical syntheses of linolenic acid esters....................22
- Synthesis of linolenic acid methyl ester (**5**)....................22
- Synthesis of linolenic acid ethyl ester (**6**)....................22

Biotransformations....................24
- Synthesis of 13-HPOT (**2**)....................24
- Synthesis cis-(+)-12-OPDA (**4**)....................26
- Optimization of the synthesis of cis-(+)-12-OPDA (**4**)....................27
- Extraction optimization....................29
- Esterification cis-(+)-12-OPDA....................31
- General procedure (GP1) Metathesis reactions....................31
- Synthesis of Ethyl 8-((1S,5S)-5-((Z)-5-hydroxyhex-2-en-1-yl)-4-oxocyclopent-2-en-1-yl)octanoate (**8**)....................32
- Synthesis of Ethyl 8-((1S,5S)-4-oxo-5-((Z)-6-oxohept-2-en-1-yl) cyclopent-2-en-1-yl)octanoate (**9**)....................32
- Synthesis of Ethyl 8-((1S,5S)-5-((Z)-6-cyanohex-2-en-1-yl)-4-oxocyclopent-2-en-1-yl)octanoate (**12**)....................33
- Synthesis of Ethyl 8-((1S,5S)-5-((Z)-6-bromohex-2-en-1-yl)-4-oxocyclopent-2-en-1-yl)octanoate (**10**)....................34
- NMR-Chromatogramms....................36

References..45

Experimental Information

Sequences and plasmid maps

pET28a-AtAOC2 _WTΔchlp(C)His

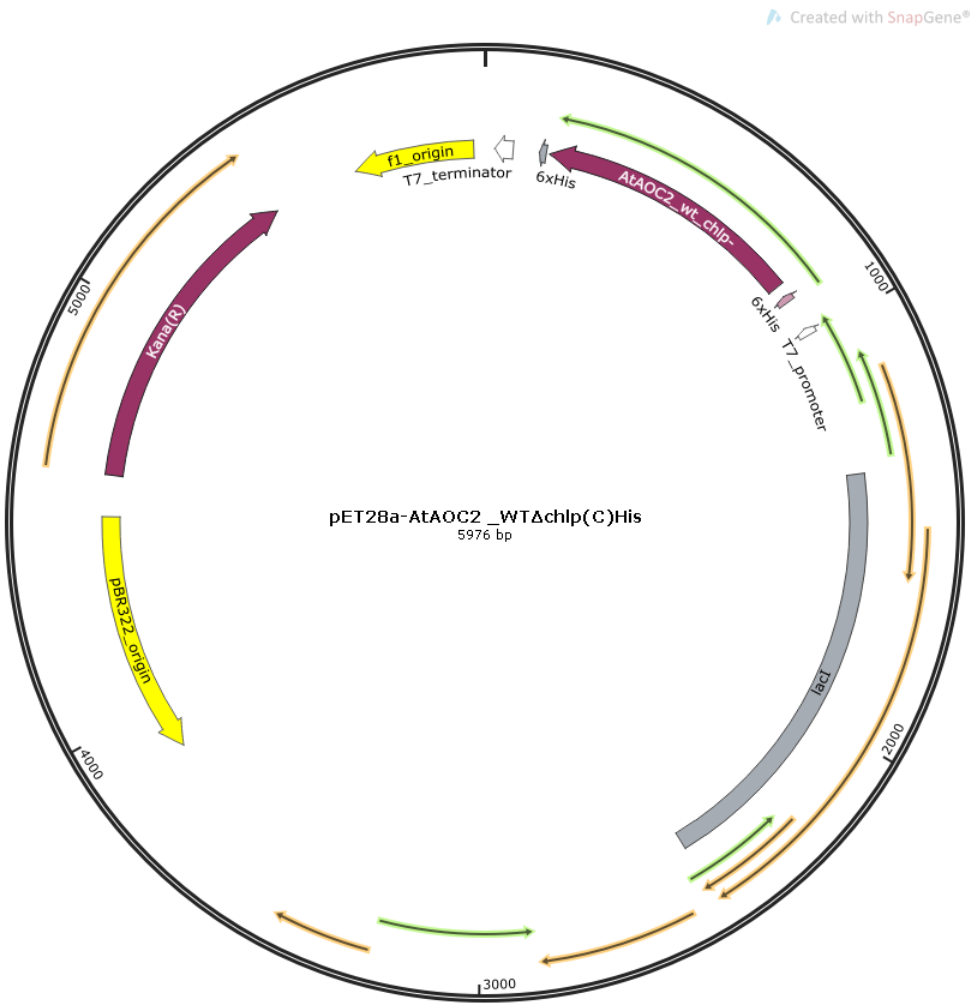

Figure 1: Allene Oxide Cyclase (AOC2) from Arabidopsis thaliana in pET28(a)+, plasmid map, without chloroplast target sequence.

Allene Oxide Cyclase (AOC2) from *Arabidopsis thaliana* wild-type sequence, without chloroplast target sequence[1].

ATGGGCAGCAGCCATCATCATCATCATCACAGCAGCGGCCTGGTGCCGCGCGGCAGC
CATATGCTTGGTTCCTCTAAATCCTTCCAAAATCTTGGTATCTCATCTAACGGTTCAGATT
TCTCCTATCCATCAAGTTTCACTGCCAAGAAGAACCTCACTGCTTCTCGAGCTCTCTCCC
AAAACGGGAATATCGAAAACCCTAGACCAAGCAAAGTTCAAGAACTGAGTGTGTACGAA
ATCAACGAATTAGATCGACACAGCCCCAAGATTCTTAAAAACGCATTCAGCTTAATGTTC
GGTCTCGGAGATCTCGTACCATTCACAAACAAACTCTACACAGGCGATCTCAAGAAACG

CGTGGGCATCACGGCAGGTCTCTGCGTCGTCATCGAACACGTCCCAGAGAAGAAAGGT
GAAAGATTCGAAGCTACTTATAGCTTCTACTTCGGAGACTATGGCCACTTGTCCGTTCAA
GGACCATACTTGACTTACGAGGATTCGTTCCTCGCCATCACTGGTGGTGCTGGAATCTT
TGAAGGTGCCTACGGACAGGTCAAGCTTCAACAGCTTGTGTATCCGACAAAACTGTTCT
ACACTTTTTACCTTAAAGGGTTGGCTAATGATTTGCCGTTGGAGCTCACCGGAACACCG
GTACCGCCGTCTAAGGACATAGAGCCGGCGCCGGAAGCTAAGGCACTGGAGCCTAGC
GGAGTTATAAGTAACTATACCAACTAA-

Amino acid-sequence

MGSSHHHHHHSSGLVPRGSHMLGSSKSFQNLGISSNGSDFSYPSSFTAKKNLTASRALSQ
NGNIENPRPSKVQELSVYEINELDRHSPKILKNAFSLMFGLGDLVPFTNKLYTGDLKKRVGIT
AGLCVVIEHVPEKKGERFEATYSFYFGDYGHLSVQGPYLTYEDSFLAITGGAGIFEGAYGQV
KLQQLVYPTKLFYTFYLKGLANDLPLELTGTPVPPSKDIEPAPEAKALEPSGVISNYTN

pQE30-AtAOC2 _WTΔchlp(N)His

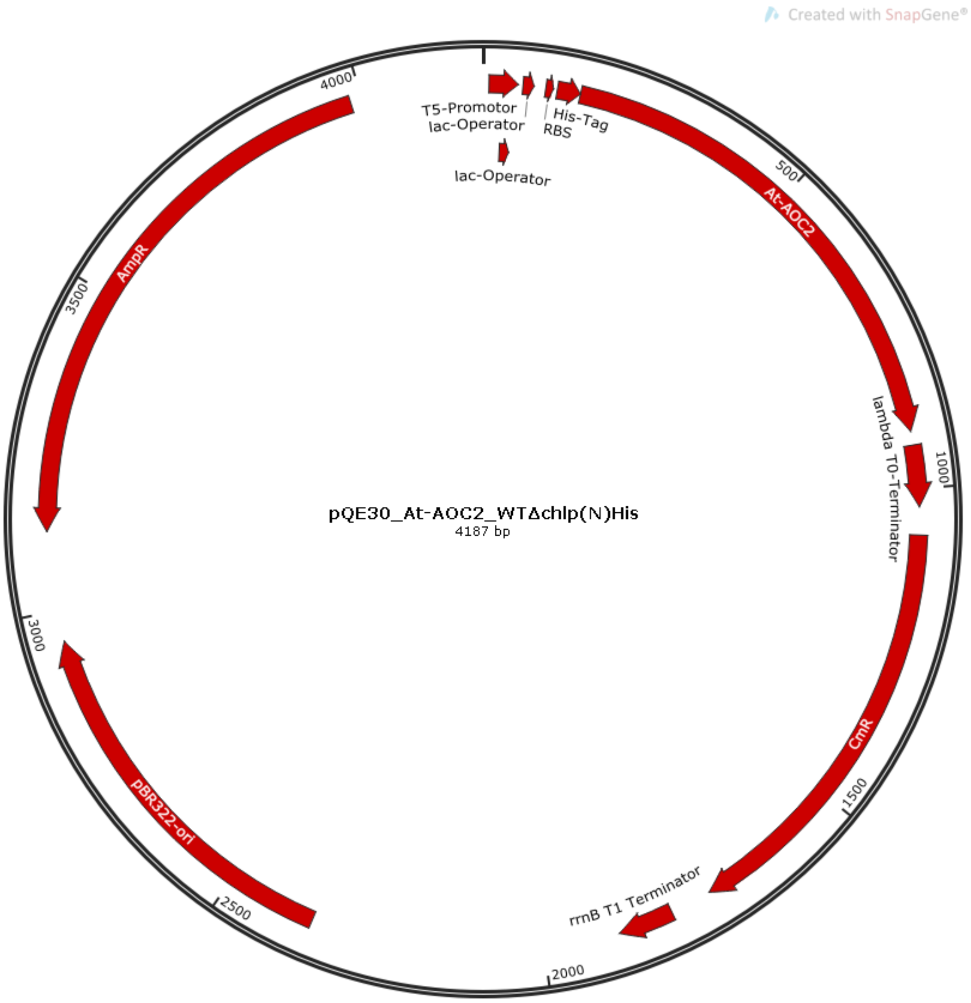

Allene Oxide Cyclase (AOC2) from *Arabidopsis thaliana* in pQE30 wild-type sequence, without chloroplast target sequence[1]

ATGAGAGGATCGCATCACCATCACCATCACGGATCCATGCTTGGTTCCTCTAAATCCTT
CCAAAATCTTGGTATCTCATCTAACGGTTCAGATTTCTCCTATCCATCAAGTTTCACTGC

CAAGAAGAACCTCACTGCTTCTCGAGCTCTCTCCCAAAACGGGAATATCGAAAACCCTA
GACCAAGCAAAGTTCAAGAACTGAGTGTGTACGAAATCAACGAATTAGATCGACACAGC
CCCAAGATTCTTAAAAACGCATTCAGCTTAATGTTCGGTCTCGGAGATCTCGTACCATTC
ACAAACAAACTCTACACAGGCGATCTCAAGAAACGCGTGGGCATCACGGCAGGTCTCT
GCGTCGTCATCGAACACGTCCCAGAGAAGAAAGGTGAAAGATTCGAAGCTACTTATAGC
TTCTACTTCGGAGACTATGGCCACTTGTCCGTTCAAGGACCATACTTGACTTACGAGGA
TTCGTTCCTCGCCATCACTGGTGGTGCTGGAATCTTTGAAGGTGCCTACGGACAGGTCA
AGCTTCAACAGCTTGTGTATCCGACAAAACTGTTCTACACTTTTTACCTTAAAGGGTTGG
CTAATGATTTGCCGTTGGAGCTCACCGGAACACCGGTACCGCCGTCTAAGGACATAGA
GCCGGCGCCGGAAGCTAAGGCACTGGAGCCTAGCGGAGTTATAAGTAACTATACCAAC
TAA

Amino acid-sequence

MRGSHHHHHHGSMLGSSKSFQNLGISSNGSDFSYPSSFTAKKNLTASRALSQNGNIENPR
PSKVQELSVYEINELDRHSPKILKNAFSLMFGLGDLVPFTNKLYTGDLKKRVGITAGLCVVIEH
VPEKKGERFEATYSFYFGDYGHLSVQGPYLTYEDSFLAITGGAGIFEGAYGQVKLQQLVYPT
KLFYTFYLKGLANDLPLELTGTPVPPSKDIEPAPEAKALEPSGVISNYTN-

pUC18-AtAOC2 _WTΔchlp(N)His

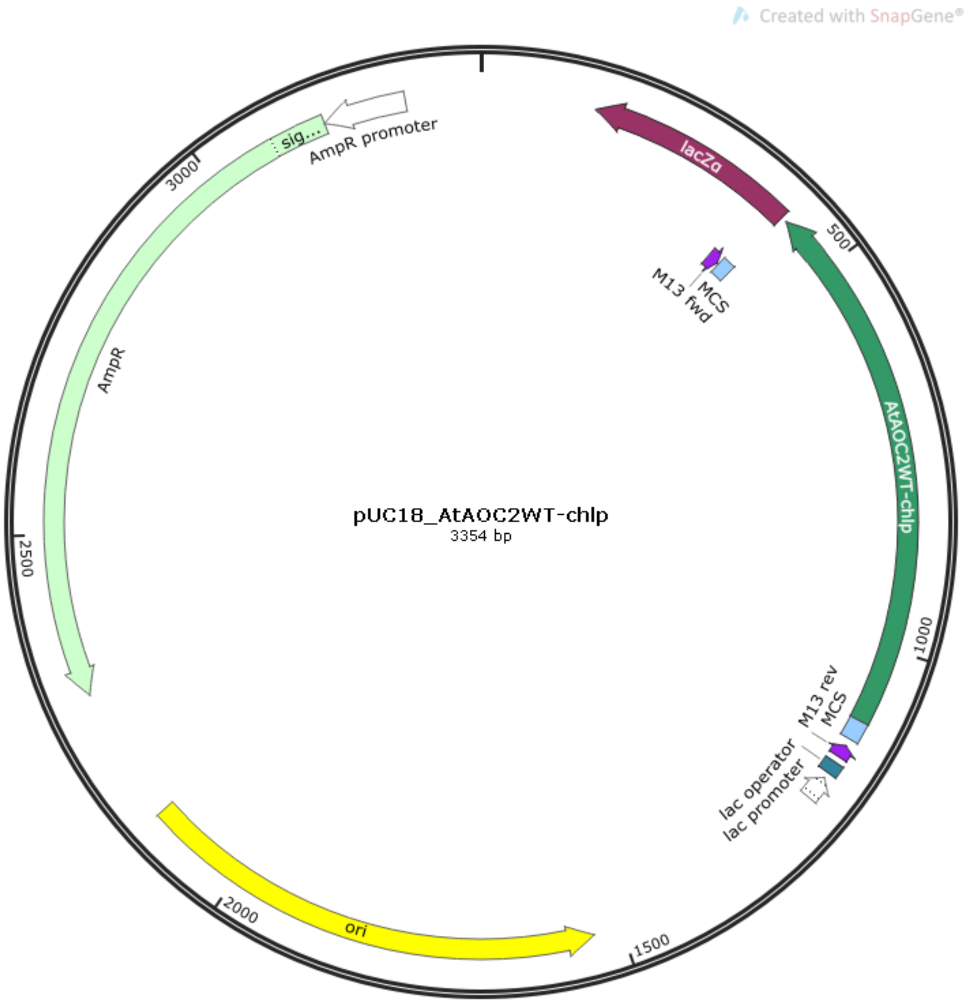

Allene Oxide Cyclase (AOC2) from *Arabidopsis thaliana* in pUC18, Nucleotide-sequence, without chloroplast target sequence

ATGAGAGGATCGCATCACCATCACCATCACGGATCCCTTGGTTCCTCTAAATCCTTCCA
AAATCTTGGTATCTCATCTAACGGTTCAGATTTCTCCTATCCATCAAGTTTCACTGCCAA
GAAGAACCTCACTGCTTCTCGAGCTCTCTCCCAAAACGGGAATATCGAAAACCCTAGAC
CAAGCAAAGTTCAAGAACTGAGTGTGTACGAAATCAACGAATTAGATCGACACAGCCCC
AAGATTCTTAAAAACGCATTCAGCTTAATGTTCGGTCTCGGAGATCTCGTACCATTCACA
AACAAACTCTACACAGGCGATCTCAAGAAACGCGTGGGCATCACGGCAGGTCTCTGCG
TCGTCATCGAACACGTCCCAGAGAAGAAAGGTGAAAGATTCGAAGCTACTTATAGCTTC
TACTTCGGAGACTATGGCCACTTGTCCGTTCAAGGACCATACTTGACTTACGAGGATTC
GTTCCTCGCCATCACTGGTGGTGCTGGAATCTTTGAAGGTGCCTACGGACAGGTCAAG
CTTCAACAGCTTGTGTATCCGACAAAACTGTTCTACACTTTTTACCTTAAAGGGTTGGCT
AATGATTTGCCGTTGGAGCTCACCGGAACACCGGTACCGCCGTCTAAGGACATAGAGC
CGGCGCCGGAAGCTAAGGCACTGGAGCCTAGCGGAGTTATAAGTAACTATACCAACTA
A

Amino acid sequence

MRGSHHHHHHGSLGSSKSFQNLGISSNGSDFSYPSSFTAKKNLTASRALSQNGNIENPRPS
KVQELSVYEINELDRHSPKILKNAFSLMFGLGDLVPFTNKLYTGDLKKRVGITAGLCVVIEHVP

EKKGERFEATYSFYFGDYGHLSVQGPYLTYEDSFLAITGGAGIFEGAYGQVKLQQLVYPTKL
FYTFYLKGLANDLPLELTGTPVPPSKDIEPAPEAKALEPSGVISNYTN-

pET28a-AtAOS _WTΔchlp(C)His

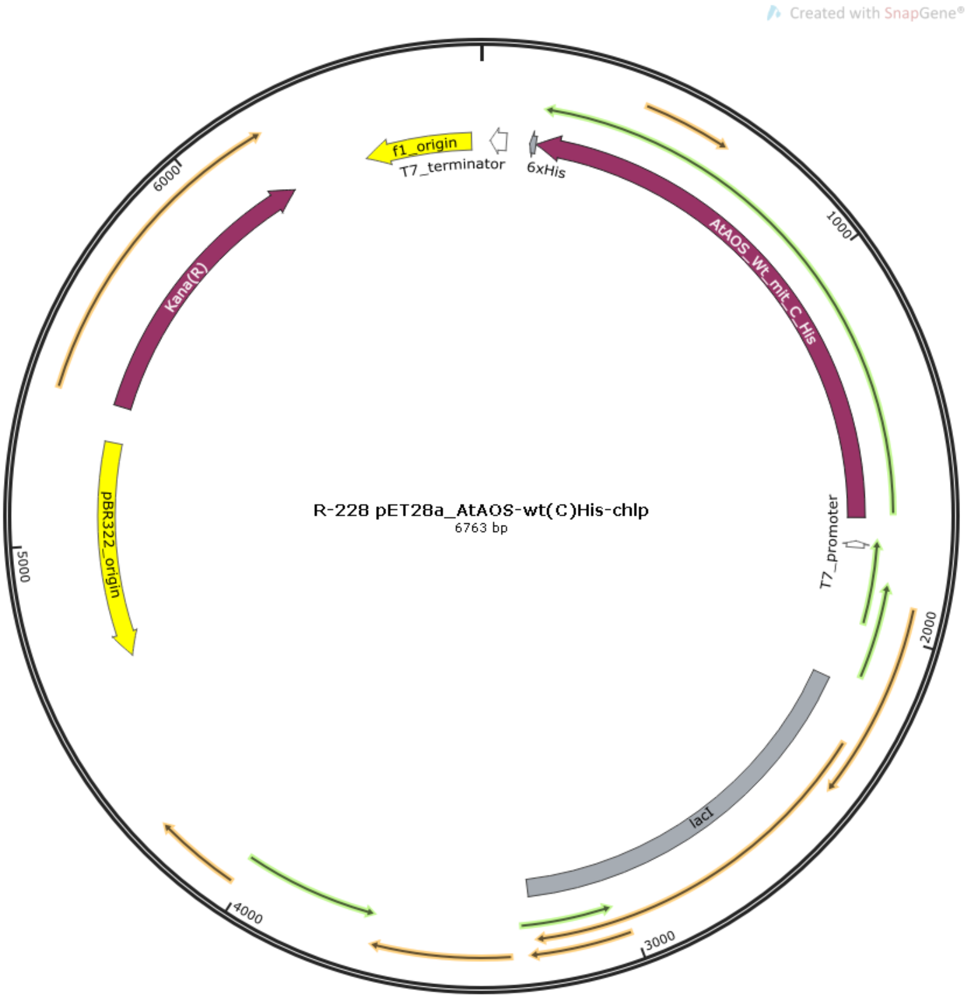

Allene Oxide Synthase (AOS) from *Arabidopsis thaliana* in pET28(a), Nucleotide-sequence, without chloroplast target sequence and solubility-sequence GCAAAAAAAACATCATCA

ATGGCAAAAAAAACATCATCAGCATCAGGATCAGAAACACCAGACCTAACAGTAGCGAC
ACGAACCGGATCCAAAGATCTCCCGATCCGAAACATACCGGGAAACTACGGTTTACCAA
TCGTAGGACCAATCAAAGACCGTTGGGATTACTTTTACGACCAAGGAGCTGAAGAGTTC
TTCAAATCACGAATCCGTAAATACAACTCCACGGTGTACAGAGTCAACATGCCACCGGG
AGCTTTTATCGCCGAGAATCCACAAGTCGTGGCTTTACTCGACGGTAAAAGCTTCCCGG
TTTTATTCGATGTCGATAAAGTCGAAAAGAAAGATCTTTTCACCGGTACTTACATGCCGT
CAACGGAACTAACCGGAGGCTACCGTATCCTCTCGTACCTCGATCCATCGGAGCCTAAA
CACGAAAAGCTCAAAAATCTCCTTTTCTTCCTCCTCAAGTCATCTCGAAACCGGATCTTC
CCTGAGTTTCAAGCTACTTACTCCGAGCTTTTCGATTCTTTGGAGAAAGAGCTTTCCCTT
AAAGGGAAAGCGGATTTCGGCGGTTCCAGCGACGGAACCGCCTTTAATTTCTTGGCTC
GGGCTTTCTACGGGACGAATCCCGCAGATACAAAGCTCAAAGCCGACGCTCCGGGTTT
GATCACTAAATGGGTTTTATTCAATCTCCATCCATTACTCTCTATTGGTTTACCGAGAGTT

ATAGAAGAACCTCTCATCCATACATTTAGTCTACCACCGGCGTTAGTCAAATCTGATTAC
CAGAGACTCTACGAGTTTTTCTTAGAATCCGCCGGTGAGATTCTCGTTGAAGCCGATAA
ATTGGGTATCTCACGAGAAGAAGCTACTCACAATCTTCTCTTCGCCACGTGCTTCAACAC
GTGGGGTGGGATGAAGATTTTGTTTCCGAATATGGTTAAACGTATCGGGCGGGCGGGT
CATCAAGTTCATAACCGATTAGCGGAGGAGATTAGATCTGTGATTAAATCCAACGGCGG
AGAACTCACGATGGGAGCGATTGAGAAAATGGAGTTAACCAAATCAGTGGTTTACGAAT
GTCTCCGGTTTGAACCACCGGTTACGGCTCAATACGGTAGAGCGAAGAAGGATCTGGT
TATCGAAAGCCACGACGCGGCGTTTAAAGTCAAAGCCGGTGAAATGCTTTACGGTTATC
AACCGTTGGCGACGAGAGATCCGAAGATTTTTGATCGGGCGGATGAGTTTGTGCCGGA
GAGATTCGTCGGAGAAGAAGGAGAGAAGCTTTTGAGGCATGTGTTGTGGTCGAATGGA
CCGGAGACGGAGACTCCGACGGTGGGGAATAAACAATGCGCCGGTAAGGATTTTGTTG
TTTTGGTGGCGAGGTTGTTTGTGATTGAGATTTTCCGGCGATATGATTCGTTTGATATTG
AGGTTGGTACGTCGCCGTTAGGAAGCTCCGTTAATTTCTCGTCGTTAAGGAAAGCTAGC
TTTGTCGACAAGCTTGCGGCCGCACTCGGGCACCACCACCACCACCACTAA

Amino acid sequence

MAKKTSSASGSETPDLTVATRTGSKDLPIRNIPGNYGLPIVGPIKDRWDYFYDQGAEEFFKS
RIRKYNSTVYRVNMPPGAFIAENPQVVALLDGKSFPVLFDVDKVEKKDLFTGTYMPSTELTG
GYRILSYLDPSEPKHEKLKNLLFFLLKSSRNRIFPEFQATYSELFDSLEKELSLKGKADFGGS
SDGTAFNFLARAFYGTNPADTKLKADAPGLITKWVLFNLHPLLSIGLPRVIEEPLIHTFSLPPA
LVKSDYQRLYEFFLESAGEILVEADKLGISREEATHNLLFATCFNTWGGMKILFPNMVKRIGR
AGHQVHNRLAEEIRSVIKSNGGELTMGAIEKMELTKSVVYECLRFEPPVTAQYGRAKKDLVI
ESHDAAFKVKAGEMLYGYQPLATRDPKIFDRADEFVPERFVGEEGEKLLRHVLWSNGPETE
TPTVGNKQCAGKDFVVLVARLFVIEIFRRYDSFDIEVGTSPLGSSVNFSSLRKASFVDKLAAA
LGHHHHHH-

For all sequences his-tags are shown in yellow, the gene itself in grey and solubility sequence in magenta.

Microbiological and biochemical procedures

Production of competent cells

From the preculture, 250 µL were isolated at transferred into a 250 mL sterile flask with 25 mL LB-Medium. When the culture reached an OD of 0.35-0.4, the cells were harvested and incubated on ice. The cells were transferred to sterile vessels and centrifuged at 5000 xg and 4˚C for 15 minutes. The cell pellet was suspended in 5 mL of calcium chloride solution (100 mM), the suspension was incubated on ice for 20 minutes. Subsequently, the suspension was centrifuged at 5000 xg and 4 ˚C for 10 minutes, the supernatant was discarded. The cells were dissolved in 500 µl of a solution of calcium chloride (100 mM) with 15% glycerol solution. The resuspension was aliquoted (100 µL) and stored at -80 °C.

Transformation of competent cells with plasmid-DNA

After digestion 10 µL plasmid DNA was added to chemical competent cells (50 µL) and incubated for 30 minutes on ice. The cells were heated at 42°C for 90 seconds and incubated again for five minutes on ice. Afterwards 1 mL of LB media was added. The mixture was heated for three hours at 37°C and 800 rpm. Subsequently, the cells were cultured on LB agar plates with suitable antibiotic and incubated overnight at 37°C.

Production of whole cell catalyst

For the production of the whole-cell catalyst, first one plasmid was transformed into *E.coli* BL21(DE3). Subsequently, competent cells were prepared from these cells. Afterwards, the other plasmid is transformed into the same *E.coli* BL21(DE3).

Heterologous expression of AtAOS and AtAOC

TB medium (400 mL) or AI medium (400 mL) with kanamycin (50 µg/mL) and ampicillin (50 µg/mL) were inoculated with 1% (v/v) overnight culture. The cultures were grown at 37 °C and 180 rpm. When the EsLeuDH-DM culture reached an OD_{600nm} of 0.6, cell cultures were induced with 400 µL of IPTG (1M). For expression the temperature was reduced to a lower temperature.

Cell Disruption

The cells were suspended in sodium phosphate buffer (50 mM, pH 7) (25% cell suspension). The cells were digested under ultrasound sonification (3x 5 minutes, 5x 10cycles, 10-20% power) on ice. Afterwards the suspension was centrifuged (15.000 rpm, 30 minutes).

Biochemical methods

SDS-PAGE Analysis

The overexpression of the enzymes was verified *via* SDS-PAGE. Therefore, the cells suspension was disrupted by ultrasound sonification on ice. Before the first centrifugation-step, a sample was taken (insoluble fraction). For the isolation of the inclusion bodies, the supernatant after the first centrifugation-step (4000 xg, 30 minutes, 4°C) was used and centrifuged again (20000 x g, 30 minutes, 4°C), the pellet contains the inclusion bodies. The 10 µL of the diluted crude extract (1 mg protein/mL) were transferred into the collection gel and analyzed *via* SDS-PAGE (15% separation gel).

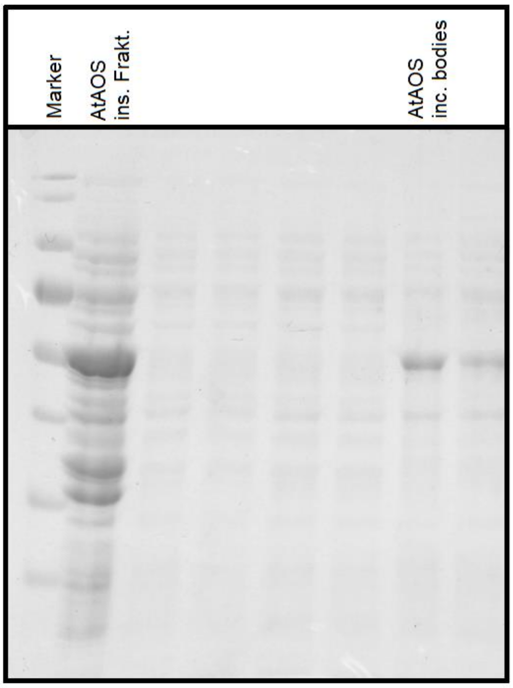

Figure 1: SDS-PAGE pET28a_AtAOS. Stained with Coomassie Brilliant Blue R-250, **lane 1**: marker (*Thermo Scientific* PageRuler® Prestained Protein Ladder); all genes were expressed in *E. coli* BL21(DE3) at 25 °C, l**ane 2**: insoluble fraction AtAOS; **lane 3**: inclusion bodies AtAOS.

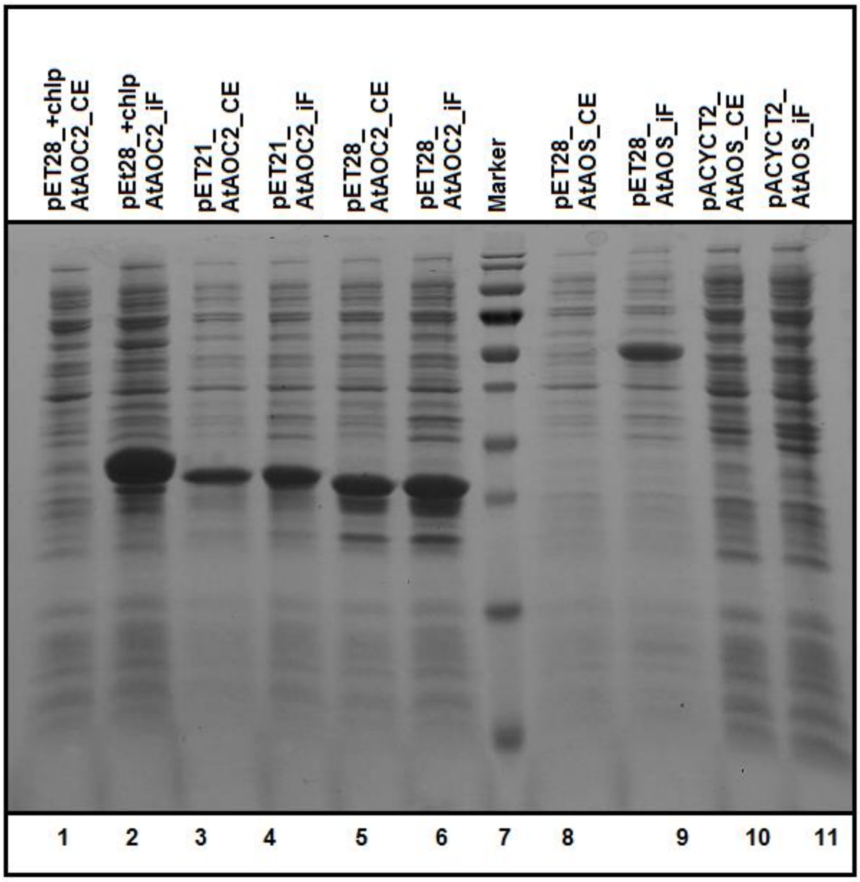

Figure 2: SDS-PAGE BL21(DE3) of AtAOS-WTΔchlp and AtAOC2-WT in different vectors. Stained with Coomassie Brilliant Blue R-250, l**ane 1**: crude extract of pET28-AtAOC2-WT with chloroplast target sequence, **lane 2**: insoluble fraction pET28-AtAOC2-WT with chloroplast target sequence, l**ane 3**: crude extract of pET21-AtAOC2-WT without chloroplast target sequence, l**ane 4**: insoluble fraction of pET21-AtAOC2-WT without chloroplast target sequence, l**ane 5**: crude extract of pET28-AtAOC2-WT without chloroplast target sequence, l**ane 6**: insoluble fraction of pET28-AtAOC2-WT without chloroplast target sequence, **lane 7**: marker (*Thermo Scientific* PageRuler® Prestained Protein Ladder), l**ane 8**: crude extract of pET28-AtAOS-WT without chloroplast target sequence, l**ane 9**: insoluble fraction of pET28-AtAOSWT without chloroplast target sequence, l**ane 8**: crude extract of pACYCT2-AtAOS-WT without chloroplast target sequence, l**ane 9**: insoluble fraction of pACYCT2-AtAOSWT without chloroplast target sequence.

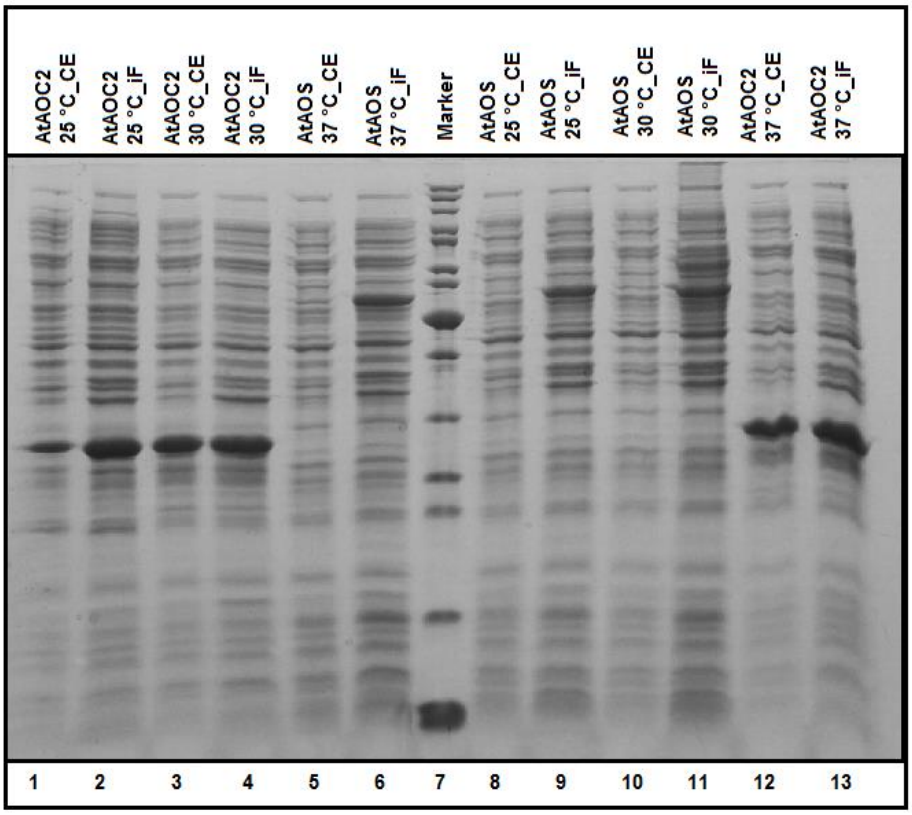

Figure 3: SDS-PAGE BL21(DE3) of AtAOS-WTΔchlp and AtAOC2-WTin pET28a, expressed at different temperatures. Stained with Coomassie Brilliant Blue R-250, l**ane 1**: crude extract of pET28-AtAOC2-WT without chloroplast target sequence, expressed at 25 °C, **lane 2**: insoluble fraction pET28-AtAOC2-WT without chloroplast target sequence expressed at 25 °C, l**ane 3**: crude extract of pET28-AtAOC2-WT without chloroplast target sequence, expressed at 30 °C, **lane 4**: insoluble fraction pET28-AtAOC2-WT without chloroplast target sequence expressed at 30 °C, l**ane 5**: crude extract of pET28-AtAOS-WT without chloroplast target sequence, expressed at 37 °C, **lane 6**: insoluble fraction pET28-AtAOS-WT without chloroplast target sequence expressed at 37 °C ,**lane 7**: marker (*Thermo Scientific* PageRuler® Unstained Protein Ladder), l**ane 8**: crude extract of pET28-AtAOS-WT without chloroplast target sequence, expressed at 25 °C, **lane 9**: insoluble fraction pET28-AtAOS-WT without chloroplast target sequence expressed at 25 °C , l**ane 10**: crude extract of pET28-AtAOS-WT without chloroplast target sequence, expressed at 30 °C, **lane 11**: insoluble fraction pET28-AtAOS-WT without chloroplast target sequence expressed at 30 °C, l**ane 12**: crude extract of pET28- AtAOC2-WT without chloroplast target sequence, expressed at 37 °C, **lane 13**: insoluble fraction pET28-AtAOC2-WT without chloroplast target sequence expressed at 37 °C .

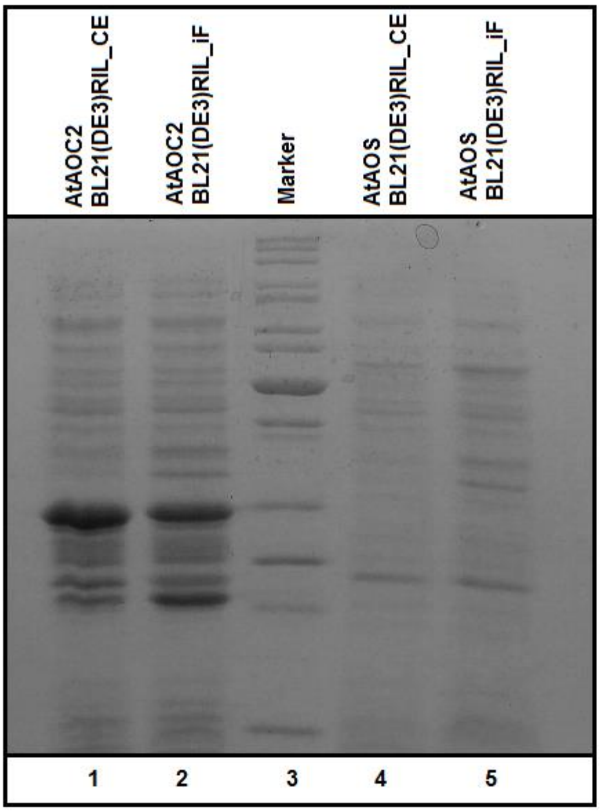

Figure 4: SDS-PAGE BL21(DE3)CodonPlus RIL of AtAOS-WTΔchlp and AtAOC2-WTin pET28a at 25 °C. Stained with Coomassie Brilliant Blue R-250, l**ane 1**: crude extract of pET28-AtAOC2-WT without chloroplast target sequence, **lane 2**: insoluble fraction pET28-AtAOC2-WT with chloroplast target sequence, **lane 3**: marker (*Thermo Scientific* PageRuler® Unstained Protein Ladder), l**ane 4**: crude extract of pET28-AtAOS-WT without chloroplast target sequence, **lane 5**: insoluble fraction pET28-AtAOS-WT with chloroplast target sequence.

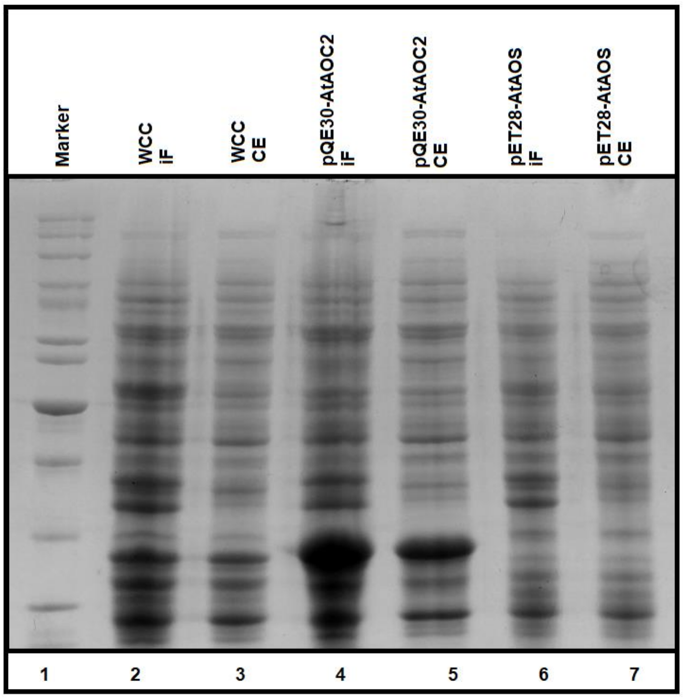

Figure 5: SDS-PAGE of expressed enzymes in BL21(DE3) at 25 °C. Stained with Coomassie Brilliant Blue R-250, l**ane 1**: marker (*Thermo Scientific* PageRuler® Unstained Protein Ladder, **lane 2**: insoluble fraction WCC consisting of pET28a-AtAOS and pQE30-AtAOC2 **lane 3**: crude extract of WCC consisting of pET28a-AtAOS and pQE30-AtAOC2, l**ane 4**: insoluble fraction of pQE30-AtAOC2, **lane 5**: crude extract of pQE30-AtAOC2, **lane 6**: insoluble fraction of pET28a-AtAOS, **lane 7**: crude extract of pET28a-AtAOS.

Activity Assay

First, an absorbance spectrum was recorded from a stock solution of 1 μL / mL purified 13-HPOT (**2**) in sodium phosphate buffer (50 mM, pH 7).

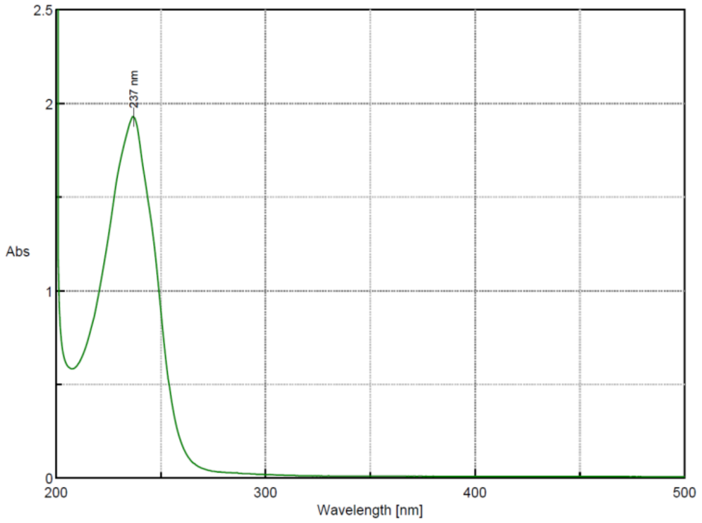

Figure 3: Absorbance spectrum for 13-HPOT.

Afterwards, 13-HPOT (**2**) was dissolved in sodium phosphate buffer (50 mM, pH 7, 1 mL) at different concentrations and the absorption was measured.

Table 1: Absorption of 13-HPOT (**2**) at different concentrations.

	**13-HPOT (2)/(mg/L)**	**Absorption Ø:**	**Extinction factor /(L/mol cm)**
**1.**	8.8	0.81	28572
	5.9	0.49	25887
	4.1	0.28	21432
**2.**	8.5	0.69	25281
	11.3	0.94	25660
	**Ø: 25383 L/mol cm**		

The extinction factor was calculated via the *Lambert-Beer* equation. The enzyme activity is defined as µmol min^{-1}·

$$U = \frac{\Delta E \cdot V_t}{t \cdot V_p \cdot d \cdot \varepsilon} F \qquad (1)$$

ΔAbs is the change in absorption, V_t is the total volume of the sample, *t* is the time and V_p is the total volume of the enzyme sample. *d* is the thickness of the cuvette and *F* is the dilution factor. ε is the extinction coefficient. In the assay for the determination of the activity of the 13-LOX, the formation of 13-HPOT (**2**) was examined at 237 nm within 60 seconds. Therefore, the extinction factor of 13-HPOT (**2**) was estimated ε= 25383 L mol^{-1} cm^{-1}. The temperature was changed from 25 to 40°C in 5°C steps. The composition of the assay is shown in the table below:

Table 2: Composition of activity assay for 13-LOX.

**Compound**	**volume/µL**	**Final**
Buffer (NH_4Cl, pH 9; 20, 50, 100 mM) (NH_4Cl, 100 mM, pH 7, 8, 9), (NaPi 100 mM), (TRIS-HCl 100 mM), (NH_4Cl, 100 mM, 0/5/10% ethanol)	890	
α-Linolenic acid (**1**) (1 mg/25 mL (Buffer))	100	0.014 µmol
13-LOX (3 mg/ 25 mL(Buffer))	20	0.0012 mg

Activity assay 13-LOX

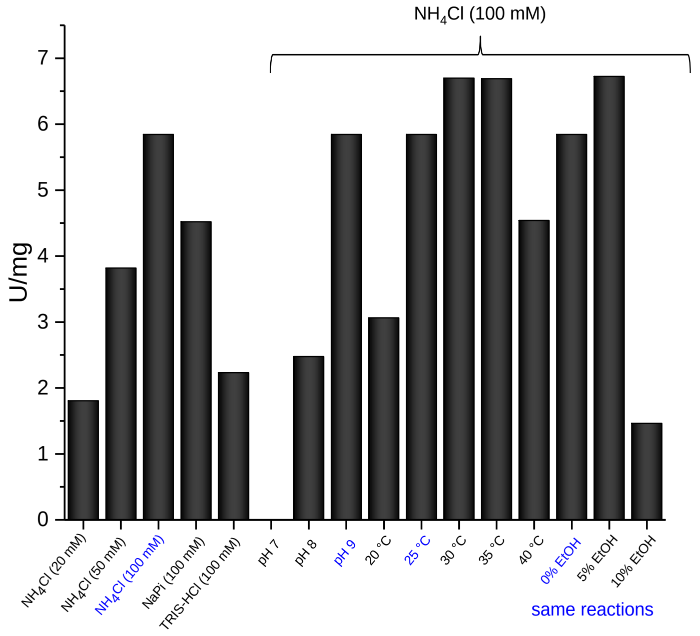

Figure 4: Activity of 13-LOX at different conditions.

Table 3: Activity of 13-LOX at different conditions.

Buffer	pH	c/mM	T/°C	EtOH/%	Abs/min	Abs/min	Abs/min	Ø(Abs/min)	U/mg
**NH_4Cl**	7	100	25	0	0.00	0.00	0.00	0.00	0.00
**NH_4Cl**	8	100	25	0	0.02	0.02	0.02	0.02	2.47
**NH_4Cl**	9	100	25	0	0.05	0.06	0.06	0.05	5.84
**NH_4Cl**	9	20	25	0	0.02	0.02		0.02	1.80
**NH_4Cl**	9	50	25	0	0.04	0.04	0.03	0.04	3.82
**NH_4Cl**	9	100	25	0	0.05	0.06	0.06	0.05	5.84
**NaPi**	9	100	25	0	0.06	0.04	0.36	0.05	4.52
**TRIS-HCl**	9	100	25	0	0.02	0.02		0.02	2.23
**NH_4Cl**	9	100	20	0	0.03	0.03		0.03	3.06
**NH_4Cl**	9	100	30	0	0.06	0.06	0.06	0.06	6.70
**NH_4Cl**	9	100	35	0	0.62	0.67		0.65	6.69
**NH_4Cl**	9	100	40	0	0.04	0.05	0.42	0.04	4.54
**NH_4Cl**	9	100	35	5	0.06	0.07	0.66	0.06	6.72
**NH_4Cl**	9	100	35	10	0.02	0.02	0.01	0.02	1.46

Activity assay 13-LOX and derivatives at different pH

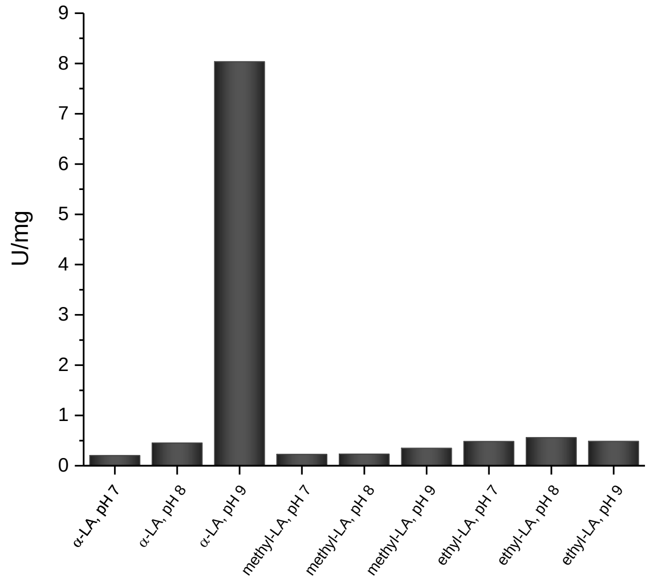

Figure 5: Activity of 13-LOX and the corresponding esters at different conditions.

Table 4: Activity of 13-LOX and the corresponding esters at different conditions.

Substrate	pH	Abs/min	Abs/min	Abs/min	Ø(Abs/min)	Abs/min	U/mg
**α-LA (1)**	7	0.00	0.00	0.00	0.00	0.19	0.20
**α-LA (1)**	8	0.00	0.01	0.00	0.00	0.43	0.45
**α-LA (1)**	9	0.06		0.10	0.08	7.63	8.03
**α-methyl-LA (5)**	7	0.00	0.00	0.00	0.00	0.21	0.22
**α-methyl-LA (5)**	8	0.00	0.00	0.00	0.00	0.22	0.23
**α-methyl-LA (5)**	9	0.00	0.01	0.00	0.00	0.33	0.35
**α-ethyl-LA (6)**	7	0.01	0.00	0.00	0.00	0.46	0.48
**α-ethyl-LA (6)**	8	0.01	0.01	0.00	0.01	0.53	0.56
**α-ethyl-LA (6)**	9	0.00	0.01	0.00	0.00	0.46	0.48

Activity assay AtAOS

The purification of the AOS by means of Ni^{2+}-NTA column chromatography was not possible; therefore, the inclusion bodies were isolated in order to test them for activity. For this purpose, some *E. coli* BL21(DE3) cells containing AtAOS were disrupted by the means of ultrasound sonification. For the isolation of the inclusion bodies, the supernatant after the first centrifugation-step (4000 xg, 30 minutes, 4°C) was used and centrifuged again (20000 xg, 30 minutes, 4 °C), the pellet contains the inclusion bodies. The pellet of the last centrifugation step was resuspended in sodium phosphate buffer (50 mM, pH 7). The protein concentration was determined *via Bradford*-assay (0.31 mg/mL).

Table 5: Composition of activity assay for AtAOS.

Compound	volume/µL
Buffer (NH_4Cl, pH 9; 20, 50, 100 mM) (NH_4Cl, 100 mM, pH 7, 8, 9), (NaPi 100 mM), (TRIS-HCl 100 mM), (NH_4Cl, 100 mM, 0/5/10% ethanol)	890
13-HPOT (**2**) (0.13 mg/25 mL (Buffer))	100
AtAOS (0.31 mg/ 1 mL(Buffer))	10

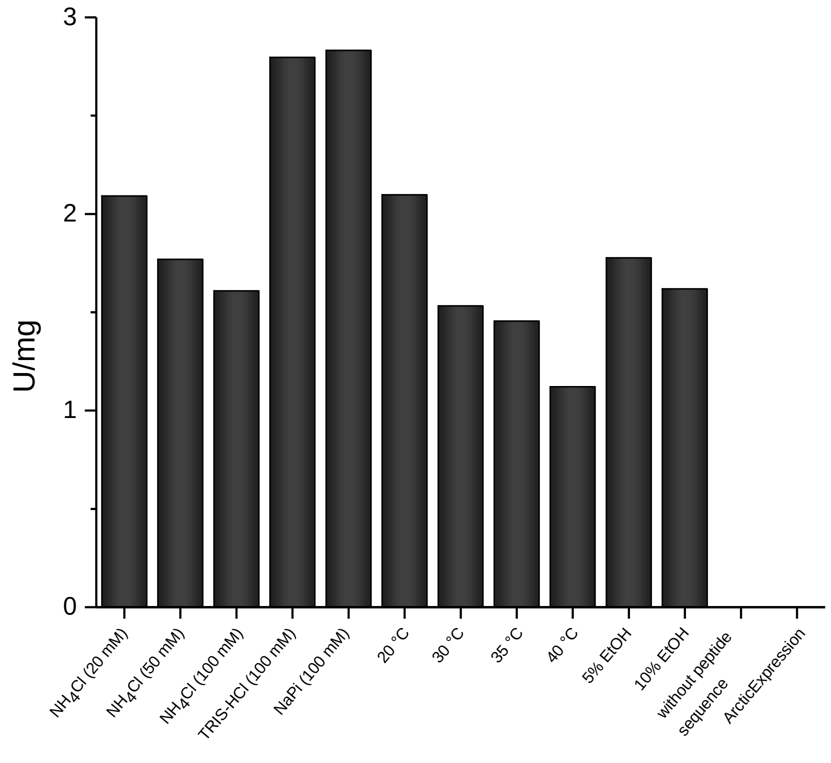

Figure 6: Activity of AtAOS at different conditions

Table 6: Activity of AtAOS at different conditions

Buffer	pH	c/mM	T/°C	EtOH/%	Abs/min	Abs/min	Abs/min	Ø(Abs/min)	U/mg
**NH_4Cl**	9	20	25	0	0.17	0.18	0.19	0.18	5.62
**NH_4Cl**	9	50	25	0	0.17	0.15	0.14	0.15	4.75
**NH_4Cl**	9	100	25	0	0.11	0.13	0.18	0.14	4.32
**TRIS-HCl**	9	100	25	0	0.21	0.24	0.27	0.24	7.52
**NaPi**	9	100	25	0	0.23	0.24	0.27	0.24	7.61
**NaPi**	9	100	25	0	0.21	0.18	0.16	0.18	5.64
**NaPi**	9	100	20	0	0.13	0.12	0.15	0.13	4.12
**NaPi**	9	100	30	0	0.13	0.15	0.10	0.13	3.91
**NaPi**	9	100	35	0	0.09	0.07	0.13	0.10	3.01
**NaPi**	9	100	40	0	0.15	0.19	0.12	0.15	4.77
**NaPi**	9	100	25	5	0.12	0.16	0.14	0.14	4.35
**NaPi**	9	100	25	10	0.17	0.18	0.19	0.18	5.62

Chemical syntheses of linolenic acid esters

*Synthesis of linolenic acid methyl ester (**5**)*

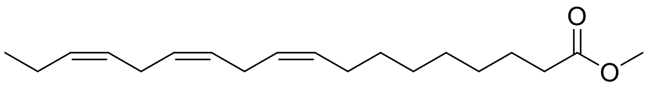

Linolenic acid methyl ester (5)
$C_{19}H_{32}O_2$
292.46 g/mol

α-Linolenic acid (**1**) (55 mg, 0.19 mmol) was dissolved in methanol (15 mL), toluene (10 mL), HCl (8% in methanol, 4°C, 3 mL) with molecular sieve (3 Å, 110 mg). The reaction was stirred for 1 h. The solvent was removed *in vacuo*. Afterwards, the crude product was extracted with water (10 mL) and *n*-hexane (10 mL). Magnesium sulfate was added to remove residual water. The solvent was removed *in vacuo.*

Yield: 57 mg, 0.12 mmol, 98%.

TLC: (R_f) = 0.81 (Cyclohexane/EtOAc/AcOH, 4.5:0.5:0.1 v/v)

^{1}H-NMR (500 MHz, $CDCl_3$) δ/ppm = 5.45 – 5.28 (m, 6H), 3.67 (s, 3H), 2.81 (ddd, ^3J = 9.3, ^2J = 4.7, 2.3 Hz, 4H), 2.31 (t, ^3J = 7.6 Hz, 2H), 2.12 – 2.01 (m, 4H), 1.62 (t, ^3J = 7.3 Hz, 2H), 1.38 - 1.24 (m, 8H), 0.98 (t, ^3J = 7.5 Hz, 3H).

The spectral data are in accordance with those given in literature.[2]

*Synthesis of linolenic acid ethyl ester (**6**)*

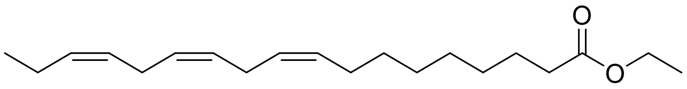

Linolenic acid ethyl ester (6)
$C_{20}H_{34}O_2$
306.49 g/mol

r

Linolenic acid (**1**, 100 mg, 0.40 mmol) were dissolved in ethanol (130 mL), toluene (20 mL), HCl (8% in ethanol, 4°C, 3 mL) with molecular sieve (4 Å, 110 mg). The reaction was stirred under reflux for 24 h. The solvent was removed *in vacuo*. Afterwards, the crude product was extracted with water (10 mL) and *n*-hexane (10 mL). Magnesium sulfate was added to remove residual water. The solvent was removed *in vacuo*.

Yield: 58 mg (0.2 mmol), 58%

TLC: (R_f) = 0.86 (Cyclohexane/EtOAc/AcOH, 4.5:0.5:0.1, v/v)

^{1}H-NMR (500 MHz, $CDCl_3$) δ/ppm = 5.37 – 5.18 (m, 6H), 4.12 (q, ^3J = 7.1, 2H), 2.81 (t, ^3J = 6.3 Hz, 4H), 2.29 (t, ^3J = 7.6 Hz, 2H), 2.11 – 2.01 (m, 4H), 1.62 (q, ^3J = 7.7 Hz, 2H), 1.31 – 1.19 (m, 8H), 1.26 (t, ^3J = 7.1 Hz, 3H), 0.98 (t, ^3J = 7.5 Hz, 3H).

^{13}C-NMR (126 MHz, $CDCl_3$) δ/ppm = 174.05, 132.10, 130.42, 128.42, 128.40, 127.86, 127.25, 60.31, 34.54, 29.72, 29.32, 29.26, 29.25, 27.35, 25.77, 25.68, 25.12, 20.70, 14.43, 14.41.

EI-MS [m/z]: 329.30 $[M+Na]^+$

HRMS (ESI) calculated for $[M+Na]^+$ 329.2451, found 329.2446

FT-IR/cm^{-1}**:** 3009 (CH- ν), 2926 (CH-ν), 1735 (C=O-ν)

Biotransformations

*Synthesis of 13-HPOT (**2**)*

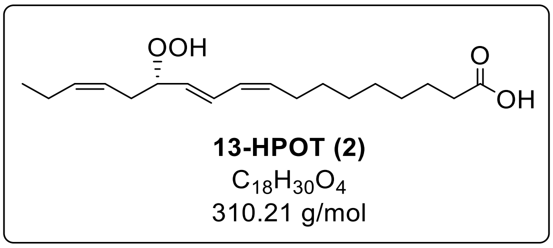

α-Linolenic acid (**1**) was diluted in ethanol (1 mL). The solution was dissolved in ammonium chloride buffer (100 mM, pH 9). Lipoxygenase from *Glycine max* was dissolved in ammonium chloride buffer (100 mM, pH 9). The reaction was performed under oxygen saturation at room temperature. Reaction was controlled *via* TLC (cyclohexane/EtOAc/AcOH 6:2:0.1 *v/v*). Afterwards the reaction mixture was acidified to pH 2 with hydrochloric acid (2 M) and extracted twice with DCM (1:1, *v/v*). Magnesium sulfate was added to remove residual water. The solvent was removed *via* rotary evaporator. The reaction compositions (which consists of either pure product or a mixture of substrate and product, but no side product) are shown in detail in the following table:

Table 7: Composition and results for the reaction towards 13-HPOT.

Experiment	NH_4Cl/mL	*α*-LA/µL	13-LOX/mg	O_2	*t*/min	Conv./%[d]
A	25	12 (0.04 mmol)	0.35	-	180	15%
B	250	120 (0.43 mol)	3.12	+	60	20%
C	250	120 (0.43 mol)	3.16	+	30	32%
D	250	120 (0.43 mol)	3.00	++	30	56%
E	250	120 (0.43 mol)	3.00	++	20	76%

[a] Reaction in an open flask; [b] Oxygen (balloon filled with oxygen) added during reaction, [c] Oxygen (balloon filled with oxygen) added before and during the reaction. **[d]** Determined by the means of ^{1}H-NMR, based on the consumption of substrate, no side products were observed

^{1}H-NMR (500 MHz, $CDCl_3$) δ/ ppm: 6.58 (ddt, *J* = 15.3, 11.1, 1.0 Hz, 1H), 5.99 (t, ^3J = 11.0 Hz, 1H;) 5.58 (dd, ^3J = 8.0 Hz ^4J = 15.2, 1H), 5.53 – 5.47 (m, 2H), 5.33 (dtt, ^3J = 7.3 Hz, ^4J = 10.7, 1.7 Hz, 1H), 4.43 (dt, ^3J = 8.2, 6.6 Hz, 1H), 2.50 – 2.44 (m, 1H), 2.34 (t, ^3J = 7.4 Hz, 2H), 2.32 – 2.28 (m, 1H), 2.18 (dtd, ^3J =7.4 Hz, ^4J =1.5, 15.0 Hz, 2H), 2.05 (pd, ^3J = 7.4 Hz, ^4J = 1.5 Hz, 2H), 1.63 (p, ^3J = 7.3 Hz, 3H), 1.25 (td, ^3J = 7.1, 4.6 Hz), 0.96 (t, ^3J = 7.5 Hz, 3H).

^{13}C-NMR (126 MHz, $CDCl_3$) δ/ ppm: 178.46, 134.53, 134.26, 130.42, 130.36, 127.68, 123.22, 86.36, 33.89, 30.76, 29.42, 29.00, 28.94, 27.80, 24.77, 20.85, 14.25.

EI-MS [m/z]: 333.26 $[M+Na]^+$349.17 $[M+K]^+$

HRMS (ESI) calculated for $[M+Na]^+$ 333.2036, found 333.2043

FT-IR/cm^{-1}**:** 3500-3000 (OH- ν), 2924 (CH- ν), 2853 (CH- ν), 1704 (C=O- ν), 1456-1409 (CH_3/CH_2-δ), 1243 (CH_3 -δ), 1200 ((*E*)-CH_2-progression bands), 948 (OH-δ).

*Synthesis cis-(+)-12-OPDA (**4**)*

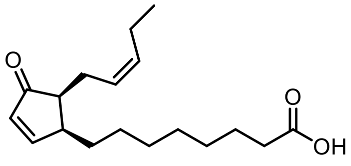

***cis*-(+)-12-OPDA (4)**
$C_{18}H_{28}O_3$
292.42 g/mol

Ammonium chloride buffer (100 mM, pH 9) was saturated with oxygen. *E.coli* BL21(DE3)CodonPlusRIL containing AOS and AOC2 were suspended in the buffer. The cell and buffer mixture was saturated with oxygen for five minutes. Lipoxygenase from *Glycine max* was dissolved in ammonium chloride buffer and dissolved in reaction mixture and saturated with oxygen for five minutes. α-Linolenic acid was dissolved in ethanol and afterwards added to the reaction mixture. The reaction was performed under oxygen saturation at room temperature. Reaction was controlled *via* TLC (cyclohexane/EtOAc/AcOH 6:2:0.1 *v/v*). After 1 h reaction time, the reaction mixture was centrifuged (10.000x g, 15 min, 4 °C). The supernatant was acidified with 2 M hydrochloric acid and extracted with DCM. Magnesium sulfate was added to remove residual water. The solvent was removed *in vacuo*, and the diastereomeric ratio of 12-OPDA (determined by comparison with literature ^{1}H-NMR data[4] for the *cis*- and *trans*-diastereomers of 12-OPDA) as well as the ratio between α-ketol and *cis*-(+)-12-OPDA were determined from the resulting crude product by means of ^{1}H-NMR spectroscopy. In all experiments the diastereomeric ratio of the *cis*-OPDA diastereomer to the *trans*-12-OPDA diastereomer was in the range of 90:10 (*cis*:*trans*) to 95:5 (*cis*:*trans*). The optical rotation measurements were done on a polarimeter "Model 341" from *Perkin Elmer*. The measurements were performed in a cuvette with a layer thickness of 1 cm at room temperature and at a wavelength of 589 nm (sodium D-line). The substances were measured as solutions with concentrations of ~1 g/100 mL in chloroform. The crude product was purified *via* automated column chromatography (MeCN/H_2O/AcOH), leading to *cis*-(+)-12-OPDA as an isolated product.

*Optimization of the synthesis of cis-(+)-12-OPDA (**4**)*

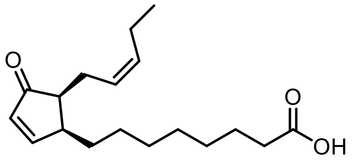

***cis*-(+)-12-OPDA (4)**
$C_{18}H_{28}O_3$
292.42 g/mol

Ammonium chloride buffer (100 mL, 100 mM, pH 9 or 500 mL, 100 mM, pH 9 for experiment 4) was saturated with oxygen. The enzymes were added as whole cells in *E.coli* BL21(DE3)CodonPlusRIL, the compositions can be taken from Table 8. AOS and AOC2 in one whole cell catalyst were used with 300 mg or 1.5 g for experiment 4. AOS and AOC2 in separate whole cells were used with 150 mg of each types of cells. The whole cells were suspended in the buffer. The cell and buffer mixture were saturated with oxygen for five minutes. Subsequently, lipoxygenase from *Glycine max* (3 mg, 40 U or 15 mg, 200 U for experiment 4), which was dissolved in ammonium chloride buffer (1 mL, 100 mM, pH 9), was added to this mixture and saturated with oxygen for five minutes. Then, the reaction was started by addition of α-linolenic acid (**1**, 100 mg, 0.36 mmol or 500 mg, 1.80 mmol for experiment 4), which was dissolved in ethanol (5 mL or 25 mL for experiment 4), to this mixture. The reaction was performed under oxygen saturation at room temperature. The reaction was controlled *via* TLC (cyclohexane/EtOAc/AcOH 6:2:0.1 *v/v*). After 1 h reaction time, the reaction mixture was centrifuged (10.000x g, 15 min, 4 °C). The supernatant was acidified with 2 M hydrochloric acid (4 mL or 20 mL for experiment 4) and extracted with DCM (3x 50 mL or 3x 250 mL for experiment 4). Magnesium sulfate was added to remove residual water. The solvent was removed *in vacuo*, and the diastereomeric ratio of 12-OPDA (determined by comparison with literature ^{1}H-NMR data[4] for the *cis*- and *trans*-diastereomers of 12-OPDA) as well as the ratio between α-ketol and *cis*-(+)-12-OPDA were determined from the resulting crude product by means of ^{1}H-NMR spectroscopy.

Table 8: Composition and results for the reaction towards *cis*-(+)-12-OPDA.

Experiment[a]	AOS	AOC2	Ratio 12-OPDA:α-Ketol
1	pET28a-AtAOS	pET28a-AtAOC2	69:31
2	pET28a-AtAOS	---	23 (racemic[b] 12-OPDA):77
3	pET28a-AtAOS	pUC18-AtAOC2	69:31
4	pET28a-AtAOS	pQE30-AtAOC2	90:10

[a] In all experiments the diastereomeric ratio of the *cis*-OPDA diastereomer to the *trans*-12-OPDA diastereomer was found to be in the range of 90:10 (*cis*:*trans*) to 95:5 (*cis*:*trans*). [b] For the 12-OPDA sample of this experiment, the measured optical rotation was as follows: α_D^{20}: 0° (c 30, $CHCl_3$).

The crude product of experiment 4 was purified *via* automated column chromatography (MeCN/H_2O/AcOH), leading to *cis*-(+)-12-OPDA (**4**) as an isolated product.

Yield of 4 (experiment 4): 31% (150 mg, 0.53 mmol)

Diastereomeric ratio of 4 (experiment 4): d.r.(*cis*:*trans*)=90:10

^{1}H-NMR (500 MHz, $CDCl_3$) δ/ppm = 7.73 (dd, ^3J = 6.0, ^4J = 2.7 Hz, 1H), 7.60 (d, ^3J = 5.7 Hz, 1H), 6.18 (dd, ^3J = 5.9, ^4J = 1.7 Hz, 1H), 6.12 (d, ^3J = 5.9 Hz, 1H), 5.46 – 5.32 (m, 2H), 2.97 (ddt, ^3J = 5.9, 10.8, 7.6, ^4J = 3.6 Hz, 1H), 2.50 (dt, ^3J = 15.3, 5.4 Hz, 1H), 2.47 – 2.41 (m, 1H), 2.35 (t, ^3J = 7.5 Hz, 2H), 2.17 – 2.10 (m, 1H), 2.06 (d, ^3J = 7.5 Hz, 2H), 1.72 (td, ^3J = 11.1, 5.0 Hz, 1H), 1.63 (p, ^3J = 7.2 Hz, 3H), 1.32 (q, ^3J = 7.1, 5.9 Hz, 8H), 1.15 (dtd, J = 14.3, 9.6, ^4J = 4.5 Hz, 1H), 0.97 (t, ^3J = 7.5 Hz, 3H).

The spectral data are in accordance with those given in literature[3,4] and according to the comparison of the ^{1}H-NMR spectroscopy data of the *cis*-diastereomer and the *trans*-diastereomer, which are reported in literature,[4] a diastereomeric ratio (*cis*:*trans*) of 90:10 was found for this experiment.

Extraction optimization

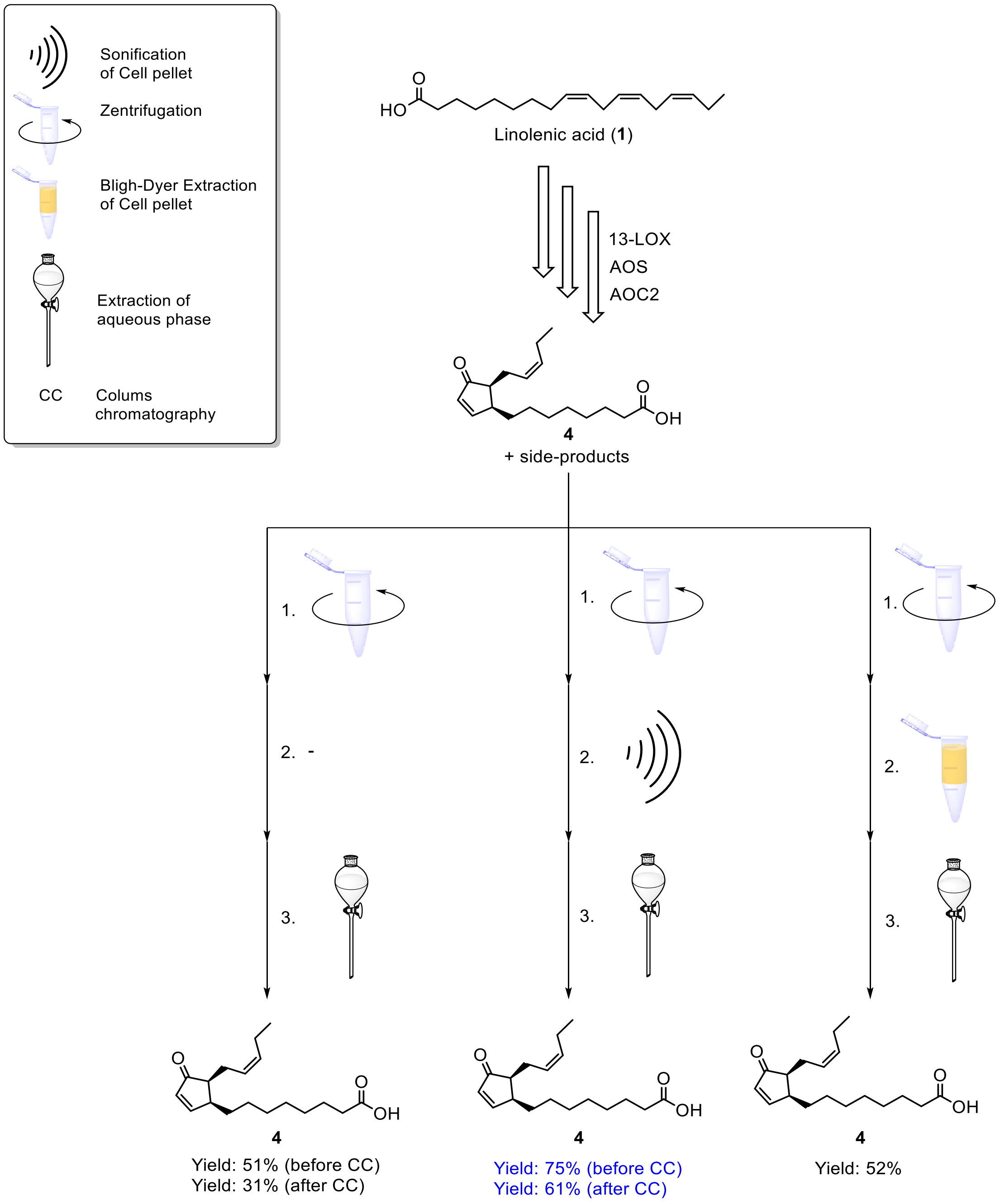

Ammonium chloride buffer (900 mL, 100 mM, pH 9) was saturated with oxygen. The enzymes were added as whole cells in *E.coli* BL21(DE3)CodonPlusRIL. AOS and AOC2 (2.7 g) were used in one whole cell catalyst. The whole cells were dissolved in the buffer. The cell and buffer mixture were saturated with oxygen for five minutes. Lipoxygenase from *Glycine max* (27 mg, 360 U) was dissolved in ammonium chloride buffer (1 mL, 100 mM, pH 9), afterwards dissolved in reaction mixture and saturated with oxygen for five minutes. α-Linolenic acid (**1**, 900 mg, 3.23 mmol) was dissolved in ethanol (45 mL) and afterwards added to the reaction

mixture. The reaction was performed under oxygen saturation at room temperature Reaction was controlled *via* TLC (cyclohexane/EtOAc/AcOH 6:2:0.1 *v/v*). After 1 h reaction time the reaction solution was divided into three parts. Each part was centrifuged (10.000x g, 15 min, 4 °C). The supernatant was acidified with 2 M hydrochloric acid and extracted with DCM (3x 50 mL or 3x 250 mL for experiment 4). Magnesium sulfate was added to remove residual water. The solvent was removed *in vacuo*. For the ultrasound approach, the cell pellet was dissolved in DCM (15 mL) and disrupted under ultrasound sonification (1x 5 minutes, 5x 10 cycles, 10-20% power), afterwards the mixture was centrifuged (10.000 xg, 5 min). The procedure was done for two times. The supernatants were combined and the solvent was removed *in vacuo*. The crude product was purified *via* automated column chromatography (MeCN/H_2O/AcOH). For the Bligh and Dyer extraction method[5], the pellet was dissolved in a mixture (MeOH, $CHCl_3$, H_2O, 2:1:1, *v/v*). After centrifugation (5000x g, 5 min) the chloroform phase was isolated and the solvent was removed *in vacuo* to furnish the desired product *cis*-(+)-OPDA (**4**).

Yield for product 4 obtained from the ultrasound method: 61% (176 mg, 0.64 mmol).

Optical rotation for product 4 obtained from the ultrasound method:

$\boldsymbol{\alpha_D^{20}}$: +104° (c 10, $CHCl_3$)

[Literature: $\boldsymbol{\alpha_D^{20}}$: +104.0° (c 9.5, $CHCl_3$)][6]

Diastereomeric ratio of 4 obtained from the ultrasound method: d.r.(*cis*:*trans*)=94:6

^{1}H-NMR for product 4 obtained from the ultrasound method (500 MHz, $CDCl_3$) δ/ppm = 7.73 (dd, ^3J = 6.0, ^4J = 2.7 Hz, 1H), 7.60 (d, ^3J = 5.7 Hz, 1H), 6.18 (dd, ^3J = 5.9, ^4J = 1.7 Hz, 1H), 6.12 (d, ^3J = 5.9 Hz, 1H), 5.46 – 5.32 (m, 2H), 2.97 (ddt, ^3J = 5.9, 10.8, 7.6, ^4J = 3.6 Hz, 1H), 2.50 (dt, ^3J = 15.3, 5.4 Hz, 1H), 2.47 – 2.41 (m, 1H), 2.35 (t, ^3J = 7.5 Hz, 2H), 2.17 – 2.10 (m, 1H), 2.06 (d, ^3J = 7.5 Hz, 2H), 1.72 (td, ^3J = 11.1, 5.0 Hz, 1H), 1.63 (p, ^3J = 7.2 Hz, 3H), 1.32 (q, ^3J = 7.1, 5.9 Hz, 8H), 1.15 (dtd, J = 14.3, 9.6, ^4J = 4.5 Hz, 1H), 0.97 (t, ^3J = 7.5 Hz, 3H).

The spectral data are in accordance with those given in literature[3,4] and according to the comparison of the ^{1}H-NMR spectroscopy data of the *cis*-diastereomer and the *trans*-diastereomer, which are reported in literature,[4] a diastereomeric ratio (*cis*:*trans*) of 94:6 was found.

Esterification cis-(+)-12-OPDA

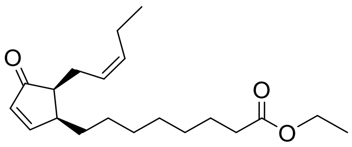

12-OPDA-ethylester (7)
$C_{20}H_{32}O_3$
320.47 g/mol

At first, *cis*-(+)-12-OPDA (**4**, 126 mg, 0.43 mmol) was dissolved in 5 mL EtOH, 770 mg molecular sieves (0.4 nm) and CAL-B (97 mg) are added. The reaction was stirred for 16 h at room temperature. The reaction was performed under oxygen saturation at room temperature. The molecular sieve is then filtered off and washed with H_2O (2x 5 mL) and extracted with *n*-hexane (2x 5 mL). The solvent was removed *in vacuo*.

Yield: 127 mg (0.39 mmol), 92%

^{1}H-NMR (500 MHz, $CDCl_3$): δ (ppm) = 7.73 (dd, ^3J= 6.0, ^4J= 2.7 Hz, 1H), 7.59 (d, ^3J= 5.7 Hz, 1H) 6.17 (dd, ^3J= 5.9, ^4J= 1.7 Hz, 1H, H3), 6.11 (d, ^3J= 5.9 Hz, 1H), 5.46 – 5.32 (m, 2H), 4.11 (q, ^3J= 7.1 Hz, 2H), 2.97 (tt, ^3J= 8.4, ^4J= 3.7 Hz, 1H), 2.49 (dd, ^3J= 5.5, ^4J= 15.3 Hz, 1H), 2.46 – 2.41 (m, 1H), 2.28 (t, ^3J= 7.5 Hz, 3H), 2.16 – 2.07 (m, 1H), 2.07 – 2.03 (m, 2H), 1.76 – 1.68 (m, 1H), 1.60 (q, ^3J= 7.1 Hz, 3H), 1.30 (s, 8H), 1.24 (t, ^3J= 7.1 Hz, 3H), 1.14 (dtd, ^4J= 18.6, 10.0, 4.6 Hz, 1H) 0.96 (t, ^3J= 7.5 Hz, 3H).

^{13}C-NMR (126 MHz, $CDCl_3$): δ (ppm)= 210.84, 173.83, 167.11, 133.90, 132.98, 132.4 , 126.98, 125.08, 60.20, 49.86, 44.29, 34.32, 30.78, 29.61, 29.42, 27.60, 24.91, 23.79, 20.80, 20.60, 14.27.

MS (ESI): m/z = 343.27 $[M+Na]^+$

HRMS (ESI) calculated for $[M+Na]^+$ 343.2244, found 343.2241

IR (neat)/cm^{-1}: 2926 (s, v, $-CH_2$), 2921 (m, v, $-CH_3$), 1730 (s, v, -C=O), 1707 (m, v, -C=C).

General procedure (GP1) Metathesis reactions

The reaction was done with *cis*-(+)-12-OPDA-ethylester (**7**, 1 eq.) and olefin (3 eq.) in degassed dichloromethane (10 mL) with *Hoveyda-Grubbs* catalyst™ 2nd generation (**C1**, 10 mol%.) for 17.5 h. Afterwards the solvent was removed, and the crude product purified *via* thin layer chromatography (cyclohexane/EtOAc *v/v* 3:1).

*Synthesis of Ethyl 8-((1S,5S)-5-((Z)-5-hydroxyhex-2-en-1-yl)-4-oxocyclopent-2-en-1-yl)octanoate (**8**)*

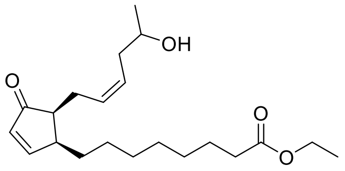

Ethyl 8-((1*S*,5*S*)-5-((*Z*)-5-hydroxyhex-2-en-1-yl)-4-oxocyclopent-2-en-1-yl)octanoate (8)
$C_{21}H_{34}O_4$
350.25 g/mol

The reaction was done with *cis*-(+)-12-OPDA-ethylester (**7**, 23.0 mg, 0.07 mmol) and 4 penten-2-ol (21.5 µL, 0.21 mmol, 3 eq.) in degassed dichloromethane (10 mL) with *Hoveyda-Grubbs* catalyst™ 2nd generation (**C1**, 4.49 mg, 0.006 mmol, 10 mol%) for 17.5 hours. Afterwards the solvent was removed, and the crude product purified *via* thin layer chromatography (cyclohexane/EtOAc *v/v* 3:1).

Yield: 6.1 mg (17 µmol), 24%

^{1}H-NMR (500 MHz, $CDCl_3$): δ (ppm)= 7.71 (ddt, ^3J= 5.8, ^4J= 11.8, 3.2 Hz, 1H), 7.59 (d, ^4J= 2.8 Hz, 1H), 6.19 – 6.14 (m, 1H), 6.13 – 6.09 (m, 1H), 5.61 (dq, ^4J= 13.2, ^3J= 6.4 Hz, 1H), 5.47 (ddt, ^3J=7.5 Hz, ^4J= 29.7, 15.0, 1H), 4.12 (q, ^3J= 7.1 Hz, 2H,), 3.83 (dt, ^4J= 12.6, ^3J= 6.4 Hz, 1H), 2.98 (m, 1H), 2.45 (dt, ^3J= 6.5, ^4J= 16.4 Hz, 1H,), 2.29 (t, ^3J= 7.5 Hz, 2H), 2.26 – 2.18 (m, 1H), 2.06 (ddd, ^3J= 6.7, ^4J= 16.0 Hz ,1H), 1.76 - 1.68 (m, 1H), 1.62 (t, ^3J= 7.3 Hz, 3H), 1.32 (m, 12H), 1.26 (t, ^3J= 7.1 Hz, 3H), 1.20 (dd, ^3J= 6.3, ^4J= 3.4 Hz, 3H).

^{13}C-NMR (126 MHz, $CDCl_3$): δ (ppm)= 204.0, 174.47, 134.78, 133.07, 131.59, 128.19, 126.56, 125.98, 67.33, 51.59, 42.69, 42.66, 34.24, 32.74, 29.49, 29.20, 29.07, 25.07, 22.77.

MS (ESI): m/z = 375.27 [M+Na]$^+$

HRMS (ESI) calculated for [M+H]$^+$ 351.25299, found 351.25366

IR (neat)/cm^{-1}: 3439 (s, v, OH) 2925 (s, ν, -CH_2), 2921 (m, ν, -CH_3), 1732 (s, ν, -C=O), 1702 (m, ν, -C=C) 1347 (w, δ, -CH_2).

*Synthesis of Ethyl 8-((1S,5S)-4-oxo-5-((Z)-6-oxohept-2-en-1-yl) cyclopent-2-en-1-yl)octanoate (**9**)*

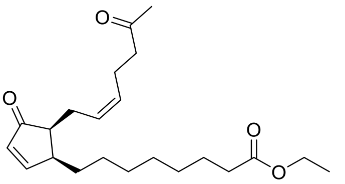

Ethyl 8-((1*S*,5*S*)-4-oxo-5-((*Z*)-6-oxohept-2-en-1-yl) cyclopent-2-en-1-yl)octanoate (9)
$C_{22}H_{34}O_4$
362.25 g/mol

The reaction was done with *cis*-(+)-12-OPDA-ethylester (**7**, 24.0 mg, 0.07 mmol) and hexen-5-one (22.0 µL, 0.22 mmol, 3 eq.) in degassed dichloromethane (10 mL) with *Hoveyda-Grubbs* Catalyst™ 2nd generation (**C1**, 4.69 mg, 0.006 mmol, 10 mol%) for 17.5 hours. Afterwards the solvent was removed, and the crude product purified *via* thin layer chromatography. 3:1).

Yield: 8.0 mg (22 µmol), 30%

^{1}H-NMR (500 MHz, $CDCl_3$): δ (ppm)= 7.72 (dd, ^{3}J = 6.0, ^{4}J= 2.9 Hz, 1H), 6.20 – 6.13 (m, 1H), 5.40 – 5.30 (m, 2H), 4.12 (q, ^{3}J= 7.1 Hz, 2H), 2.97 (m, 1H), 2.50 (m, 1H), 2.46 (dt, ^{4}J= 14.3, ^{3}J= 7.1 Hz, 1H), 2.32 (t, ^{3}J= 6.4 Hz, 2H), 2.28 (t, ^{3}J= 7.4 Hz, 2H), 2.14 (s, 1H), 2.12 (s, 3H), 2.03 (dq, ^{4}J = 24.9, ^{3}J= 7.9 Hz, 2H), 1.61 (m, 4H), 1.31 (s, 8H), 1.25 (t, ^{3}J= 7.1 Hz, 3H)

^{13}C-NMR (126 MHz, $CDCl_3$): δ (ppm)= 210.62, 208.49, 173.96, 167.19, 132.92, 132.57, 131.46, 129.60, 129.18, 127.66, 60.34, 51.50, 49.42, 46.80, 44.43, 43.47, 34.46, 30.11, 26.80, 25.05, 21.71, 14.41.

MS (ESI): m/z = 385.30 $[M+Na]^+$

HRMS (ESI) calculated for $[M+H]^+$ 363.25299, found 363.25271

IR (neat)/cm^{-1}: 2925 (s, ν, CH_2), 2854 (m, ν, CH_2), 1715 (vs, ν, C=O-Ester), 1586 (w, ν, C=C).

*Synthesis of Ethyl 8-((1S,5S)-5-((Z)-6-cyanohex-2-en-1-yl)-4-oxocyclopent-2-en-1-yl)octanoate (**12**)*

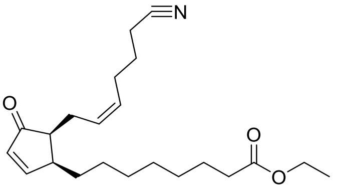

Ethyl 8-((1*S*,5*S*)-5-((*Z*)-6-cyanohex-2-en-1-yl)-4-oxocyclopent-2-en-1-yl)octanoate (12)
$C_{22}H_{33}NO_3$
359.51 g/mol

The reaction was done with *cis*-(+)-12-OPDA-ethylester (**7**, 24.0 mg, 0.06 mmol) and hex-5-enenitril (21.7 µL, 0.19 mmol, 3 eq.) in degassed dichloromethane (10 mL) with *Hoveyda-Grubbs* Catalyst™ 2nd generation (**C1**, 3.80 mg, 0.006 mmol, 10 mol%) for 17.5 hours.

Afterwards the solvent was removed, and the crude product purified *via* thin layer chromatography (cyclohexane/EtOAc *v/v* 3:1).

Yield: 9.0 mg (25 µmol), 40%

^{1}H-NMR (500 MHz, $CDCl_3$): δ (ppm)= 7.72 (dd, ^3J= 5.9, ^4J= 2.7 Hz, 1H), 6.16 (d, ^3J= 5.9 Hz, 1H), 5.47 – 5.38 (m, 2H), 4.11 (q, ^3J= 7.2 Hz, 2H), 3.61 (m, 2H), 2.98 (m, 1H), 2.60 (q, ^3J= 8.4 Hz, 2H), 2.49 (m, 1H), 2.43 (m, 1H), 2.33 (t, ^3J= 7.0 Hz, 2H), 2.28 (t, ^3J= 7.4 Hz, 2H), 2.18 (q, ^3J= 6.6 Hz, 1H), 1.72 (dq, ^4J=15.1, ^3J= 7.3 Hz, 2H), 1.61 (t, ^3J= 7.0 Hz, 2H) 1.33 (s, 8H), 1.25 (t, ^3J= 7.2 Hz, 3H).

^{13}C-NMR (126 MHz, $CDCl_3$): δ (ppm)= 210.86, 173.85, 167.15, 167.12, 132.43, 127.15, 126.97, 67.21, 60.20, 53.42, 49.45, 34.33, 30.76, 29.60, 29.55, 29.09, 28.66, 27.57, 25.68, 24.91, 14.26.

MS (ESI): m/z = 382.30 $[M+Na]^+$

HRMS (ESI) calculated for $[M+Na]^+$ 382.23526, found 382.2348

IR (neat)/cm^{-1}: 2925 (s, ν, CH_2), 2854 (m, ν, CH_2), 2250 (w, ν, C≡N), 1732 (s, ν, C=O-ester), 1702 (vs, ν, C=O-carbonyl), 1586 (w, ν, C=C)

*Synthesis of Ethyl 8-((1S,5S)-5-((Z)-6-bromohex-2-en-1-yl)-4-oxocyclopent-2-en-1-yl)octanoate (**10**)*

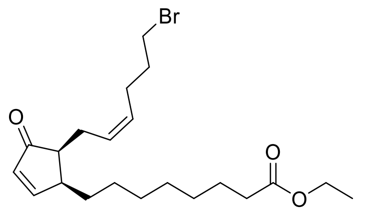

Ethyl 8-((1*S*,5*S*)-5-((*Z*)-6-bromohex-2-en-1-yl)-4-oxocyclopent-2-en-1-yl)octanoate (10)
$C_{21}H_{33}BrO_3$
413.40 g/mol

The reaction was done with *cis*-(+)-12-OPDA-ethylester (**7**, 19.4 mg, 0.06 mmol or 30.0 mg, 0.09 mmol) and 5-bromopentene (21.5 µL, 0.18 mmol, 3 eq. or 41.0 µL, 0.34 mmol, 4 eq.) in degassed dichloromethane (10 mL) with *Hoveyda-Grubbs* Catalyst™ 2nd generation (**C1**, 3.79 mg, 0.006 mmol, 10 mol% or **C1**, 13.3 mg, 0.016 mmol, 18 mol%) for 17.5 hours. Afterwards the solvent was removed, and the crude product purified *via* thin layer chromatography (cyclohexane/EtOAc *v/v* 3:1).

Yield: 10 mg (24 µmol), 40% or 23 mg (56 µmol), 61%

^{1}H-NMR (500 MHz, $CDCl_3$): δ (ppm)= 7.72 (dd, ^{3}J= 5.8, ^{4}J= 2.7 Hz, 1H), 6.16 (d, ^{3}J= 5.8 Hz, 1H), 5.61 – 5.36 (m, 2H), 4.11 (q, ^{3}J= 7.2 Hz, 2H), 3.38 (dt, ^{4}J= 15.1, ^{3}J= 6.6 Hz, 2H), 3.01 – 2.94 (m, 1H), 2.60 (m, 2H), 2.50 (m, 1H), 2.47 – 2.36 (m, 1H), 2.28 (t, ^{3}J= 7.5 Hz, 2H), 2.20 - 2.15 (m, 1H), 1.90 (m, 2H), 1.77 – 1.68 (m, 1H), 1.67 – 1.58 (m, 2H), 1.31 (s, 8H),1.25 (t, ^{3}J= 7.1 Hz, 4H).

^{13}C-NMR (126 MHz, $CDCl_3$): δ (ppm)= 210.73, 173.96, 167.18, 132.61, 130.18, 129.59, 60.34, 49.43, 44.46, 34.47, 33.34, 32.42, 31.04, 30.97, 29.75, 29.30, 29.21, 28.88, 27.73, 25.05, 14.42.

MS (ESI): m/z = 435.229 m/z $[M+Na]^+$, 437.186 m/z $[M+Na]^+$.

HRMS (ESI) calculated for $[M+Na]^+$ 435.1505, found 435.1506

IR (neat)/cm^{-1}: 2925 (s, ν, CH_2), 2854 (m, ν, CH_2), 1732 (s, ν, C=O-Ester), 1702 (vs, ν, C=O-carbonyl), 1586 (w, ν, C=C), 600 (w, ν, C-Br).

NMR-Chromatogramms

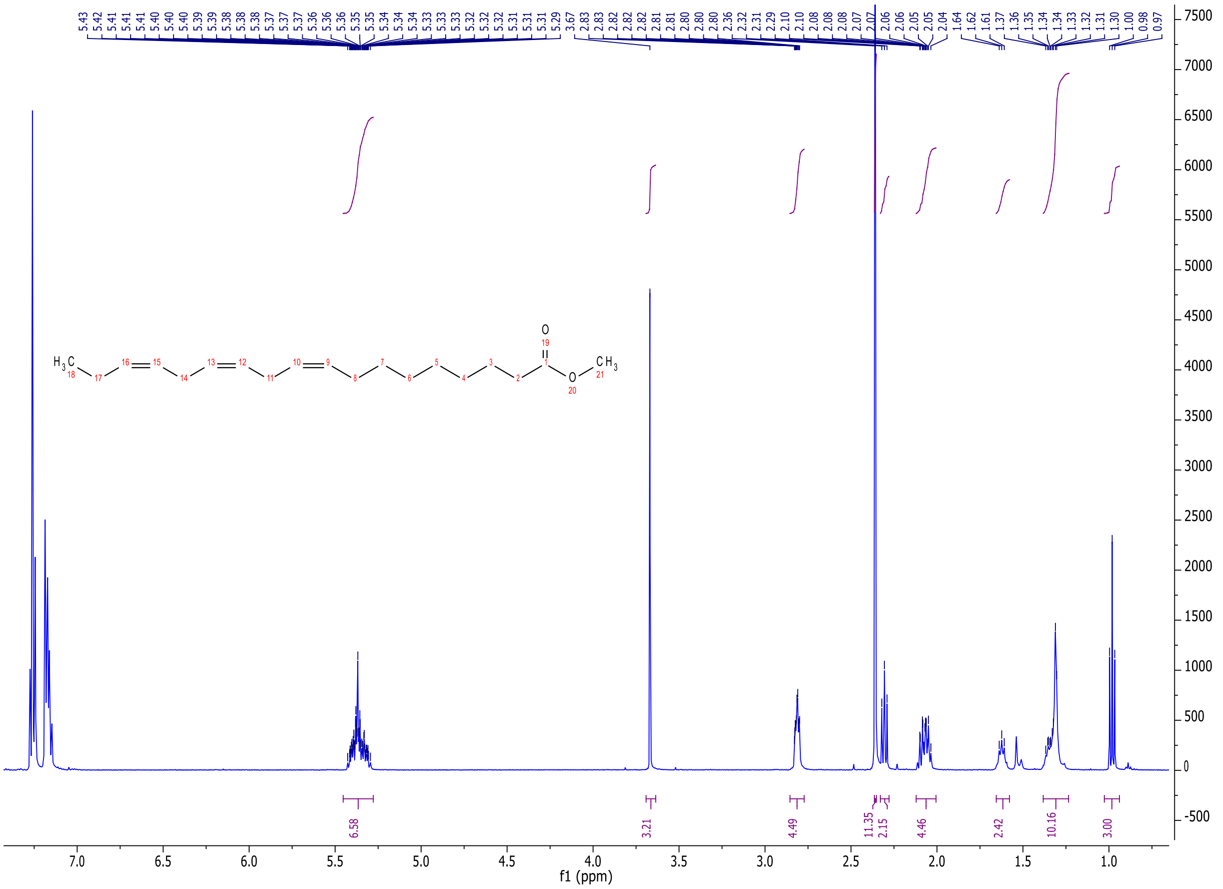

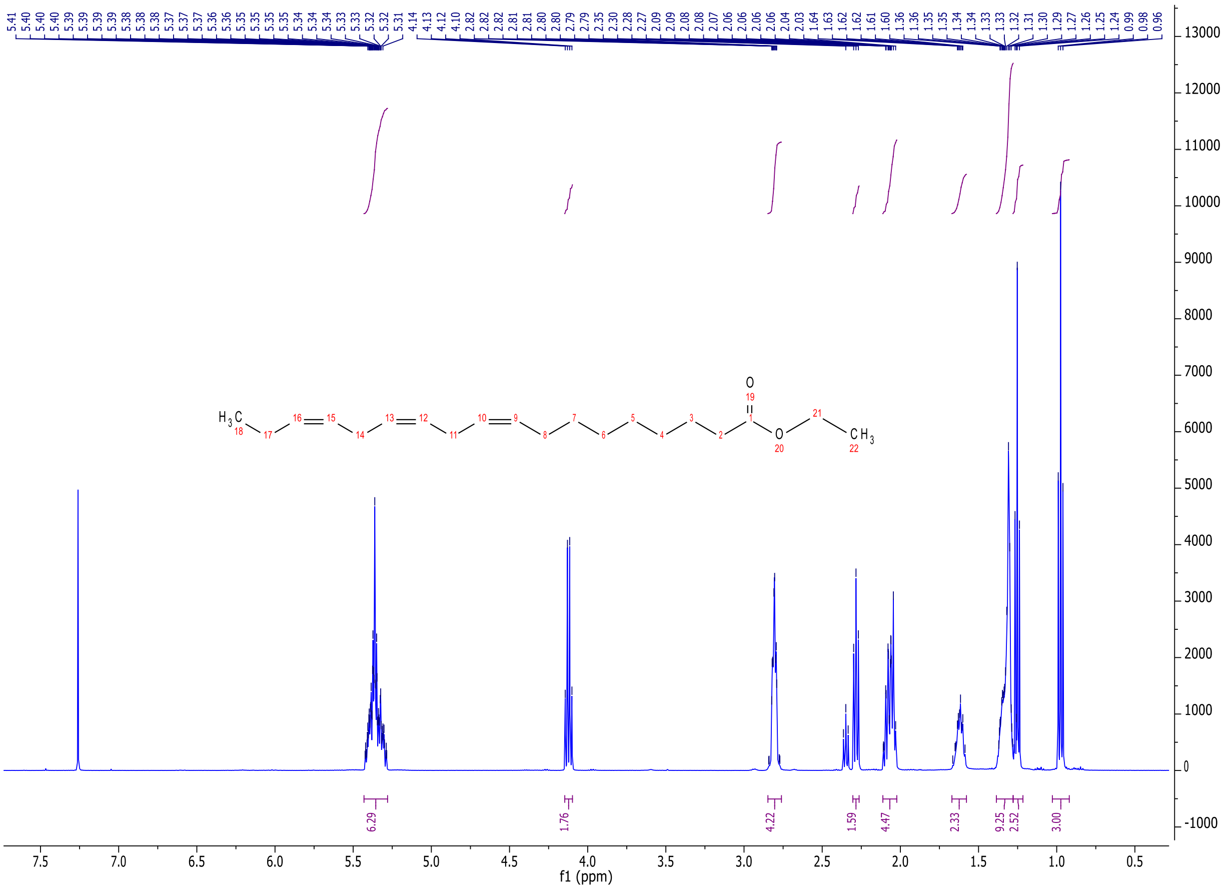
f1 (ppm)

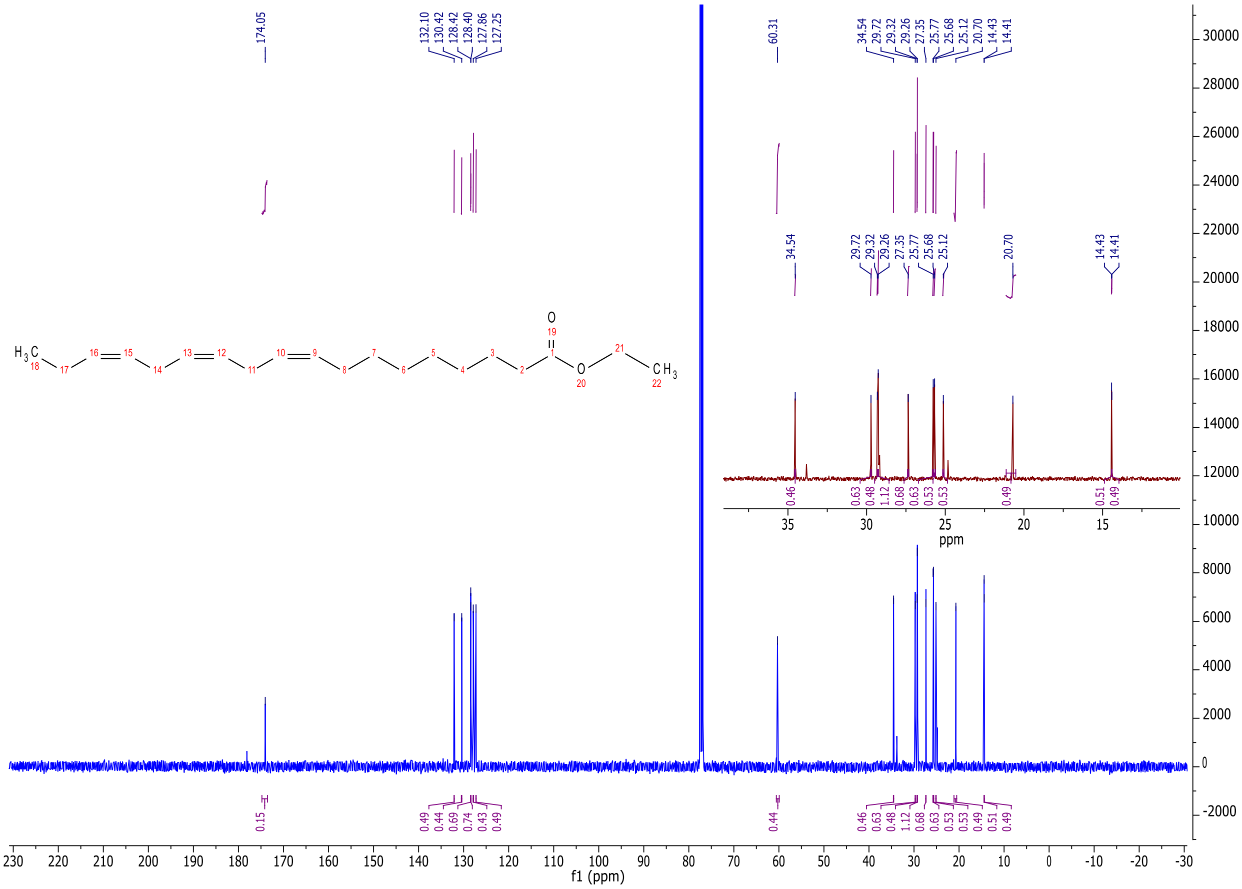
f1 (ppm)

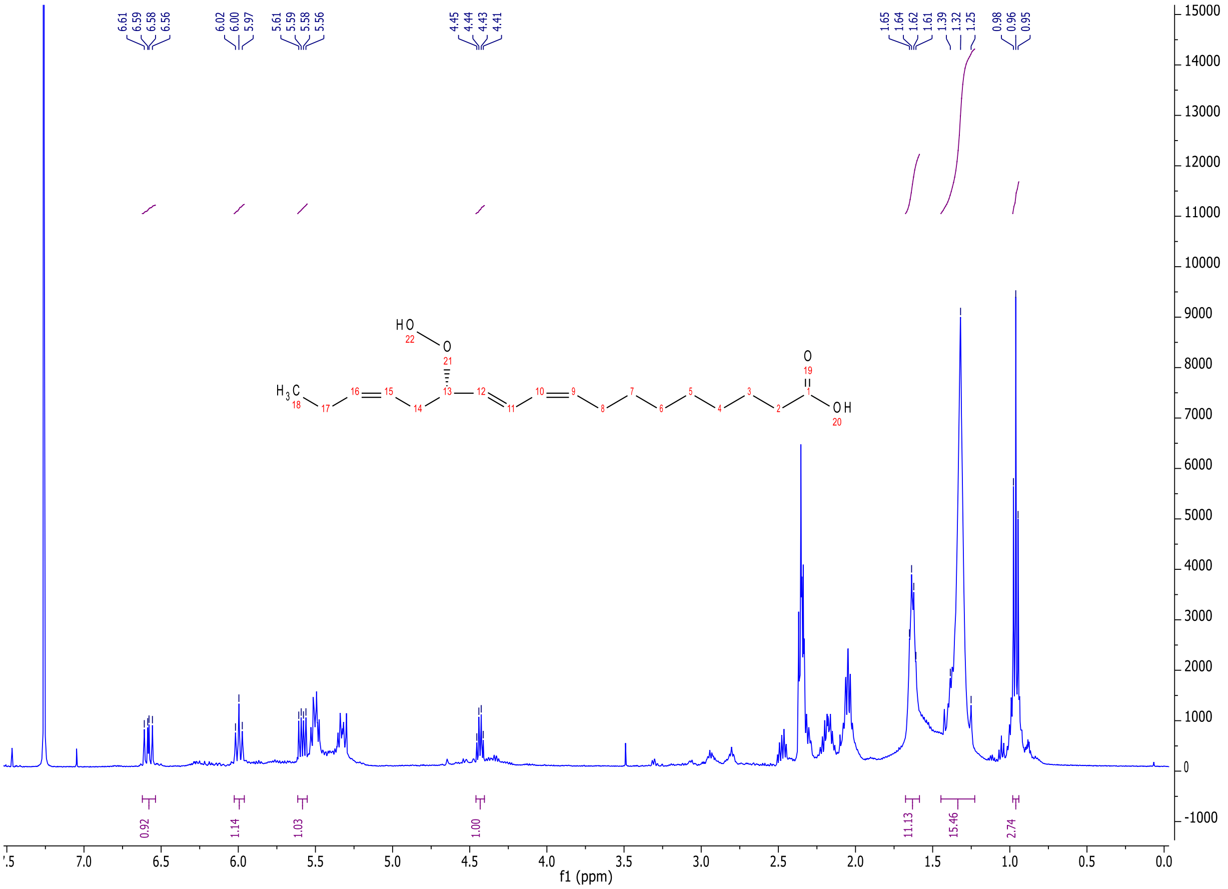
6.61
6.59
6.58
6.56
6.02
6.00
5.97
5.61
5.59
5.58
5.56
4.45
4.44
4.43
4.41
1.65
1.64
1.62
1.61
1.39
1.32
1.25
0.98
0.96
0.95
0.92
1.14
1.03
1.00
11.13
15.46
2.74
f1 (ppm)

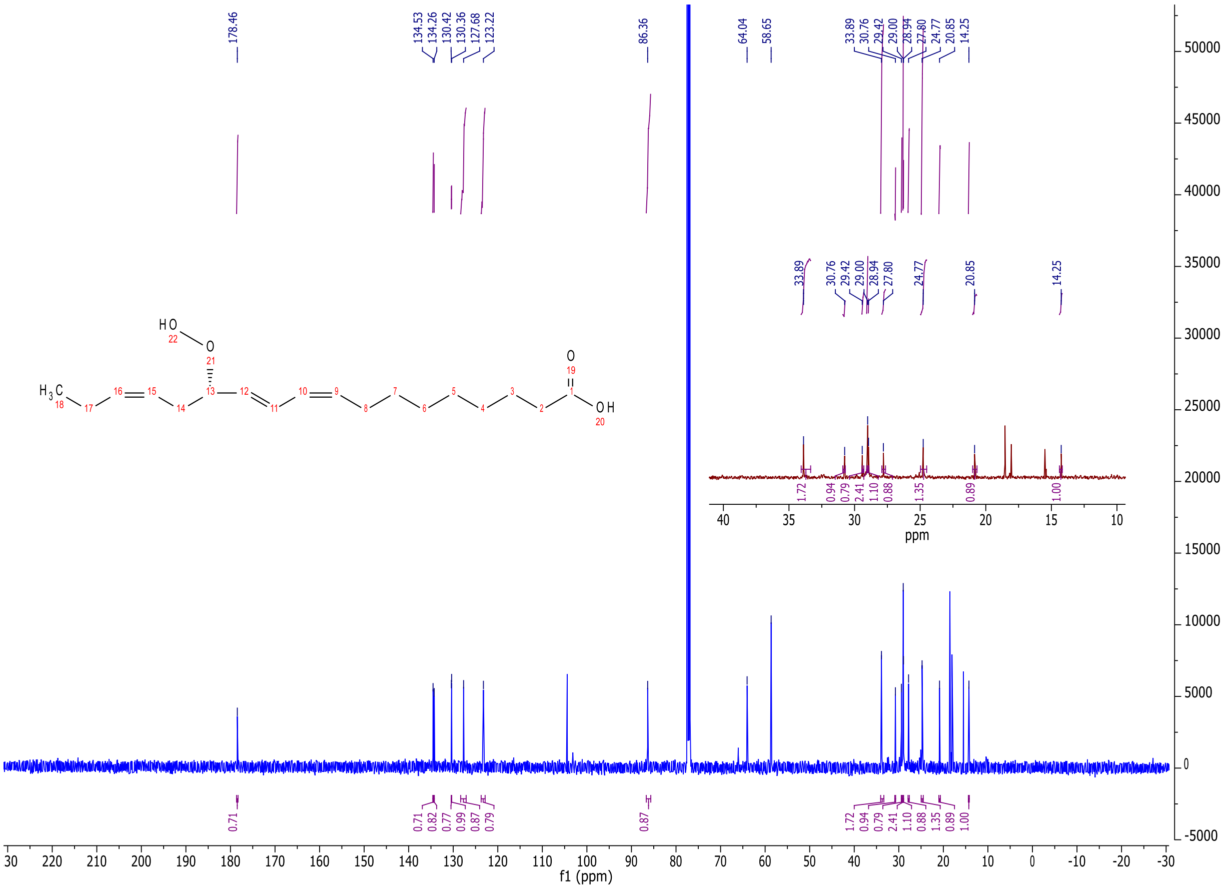
178.46
134.53
134.26
130.42
130.36
127.68
123.22
86.36
64.04
58.65
33.89
30.76
29.42
29.00
28.94
27.80
24.77
20.85
14.25
ppm
f1 (ppm)

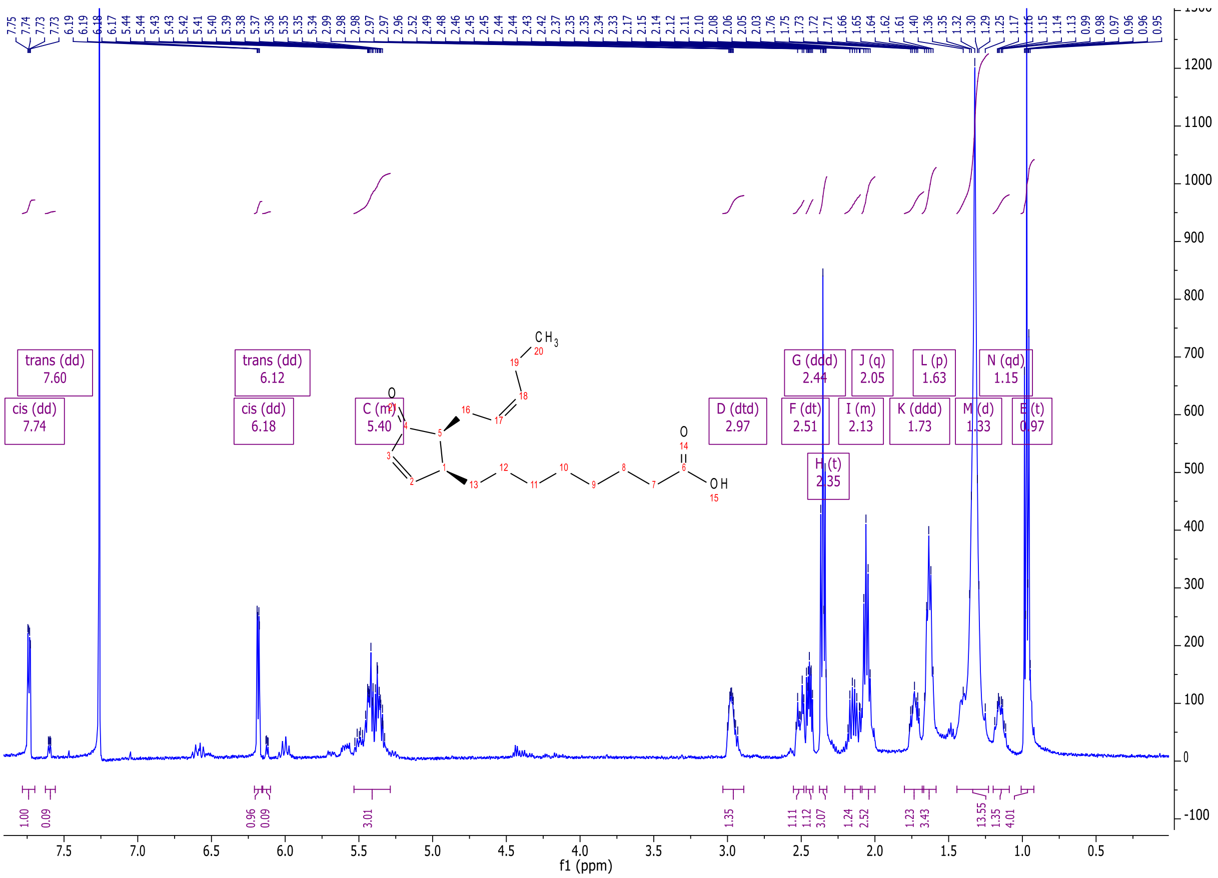
trans (dd)
7.60
cis (dd)
7.74
trans (dd)
6.12
cis (dd)
6.18
C (m)
5.40
D (dtd)
2.97
G (ddd)
2.44
F (dt)
2.51
H (t)
2.35
J (q)
2.05
I (m)
2.13
L (p)
1.63
K (ddd)
1.73
N (qd)
1.15
M (d)
1.33
E (t)
0.97
f1 (ppm)

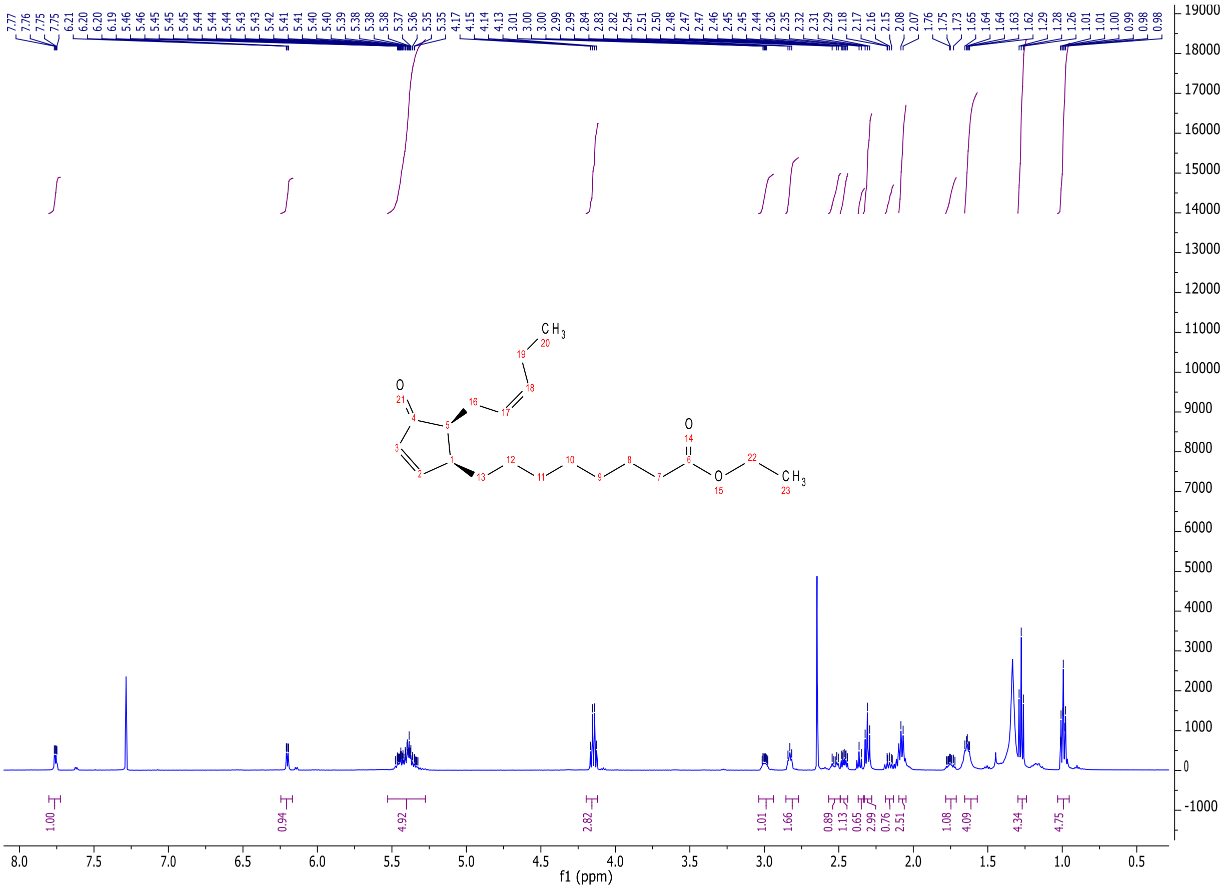
7.77 7.76 7.75 7.75 6.21 6.20 6.19 5.46 5.46 5.45 5.45 5.45 5.44 5.44 5.44 5.43 5.43 5.42 5.41 5.41 5.40 5.40 5.39 5.38 5.38 5.38 5.37 5.36 5.35 5.35 4.17 4.15 4.14 4.13 3.01 3.00 3.00 2.99 2.99 2.84 2.83 2.82 2.54 2.51 2.50 2.48 2.47 2.47 2.46 2.45 2.45 2.44 2.36 2.35 2.32 2.31 2.29 2.18 2.17 2.16 2.15 2.08 2.07 1.76 1.75 1.73 1.65 1.64 1.64 1.63 1.62 1.29 1.28 1.26 1.01 1.01 1.00 0.99 0.98 0.98
1.00 0.94 4.92 2.82 1.01 1.66 0.89 1.13 0.65 2.99 0.76 2.51 1.08 4.09 4.34 4.75
f1 (ppm)

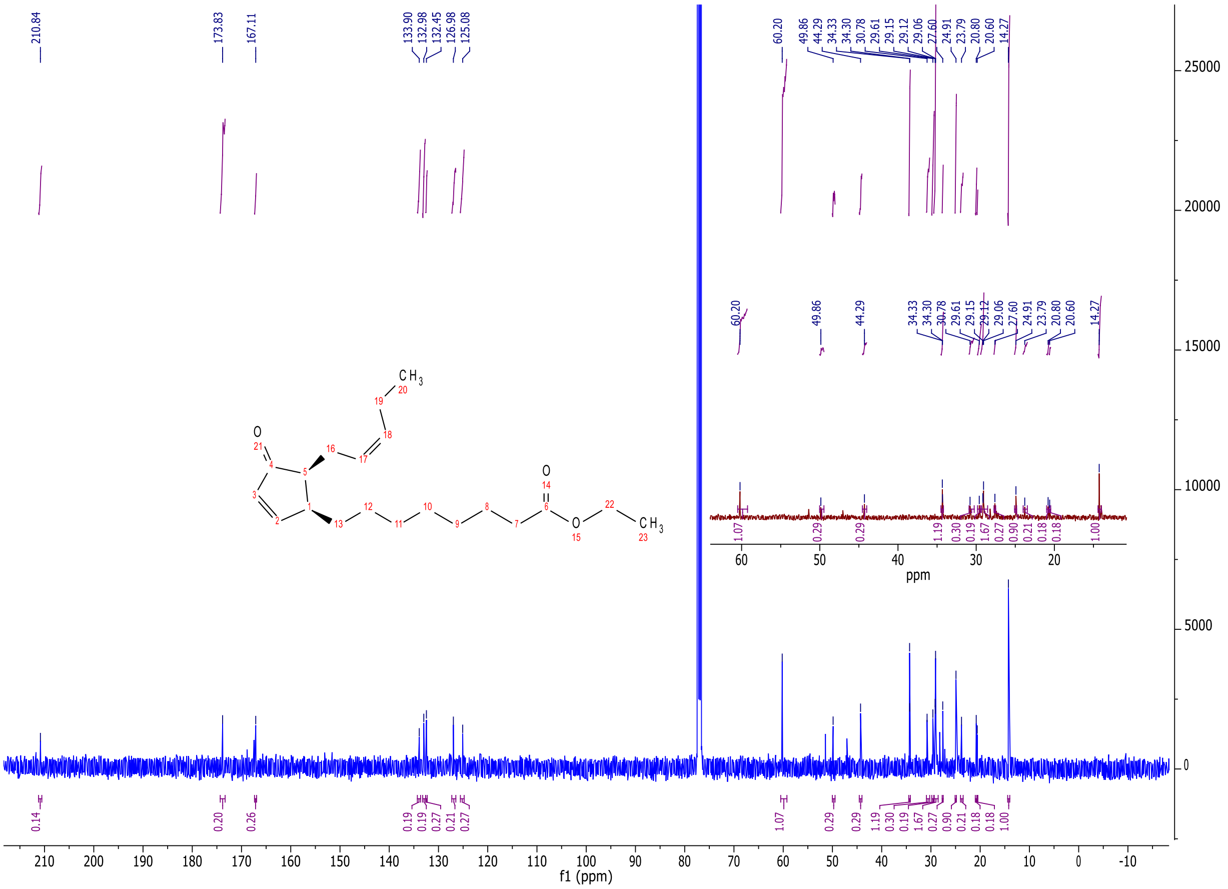
210.84 173.83 167.11 133.90 132.98 132.45 126.98 125.08 60.20 49.86 44.29 34.33 34.30 30.78 29.61 29.15 29.12 29.06 27.60 24.91 23.79 20.80 20.60 14.27
0.14 0.20 0.26 0.19 0.19 0.27 0.21 0.27 1.07 0.29 0.29 1.19 0.30 0.19 1.67 0.27 0.90 0.21 0.18 0.18 1.00
ppm
f1 (ppm)

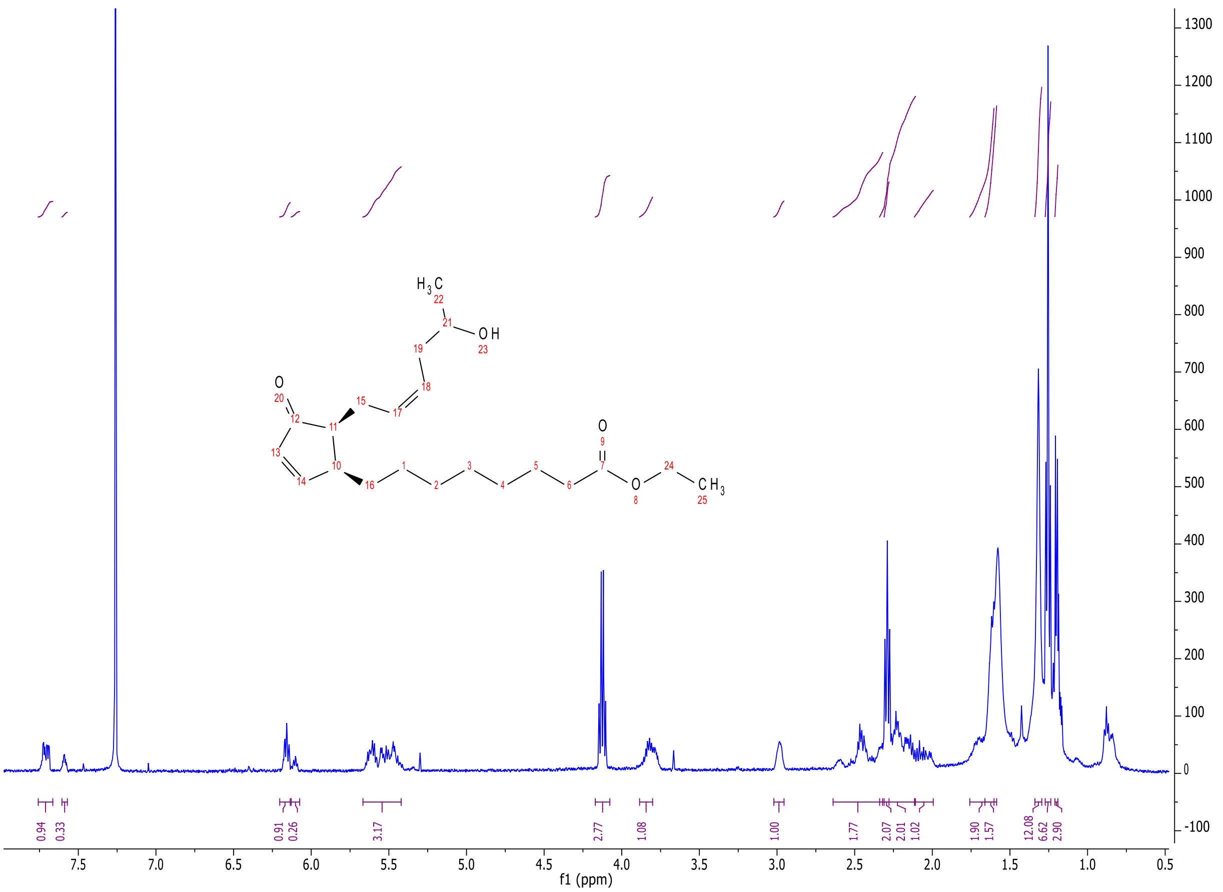
f1 (ppm)

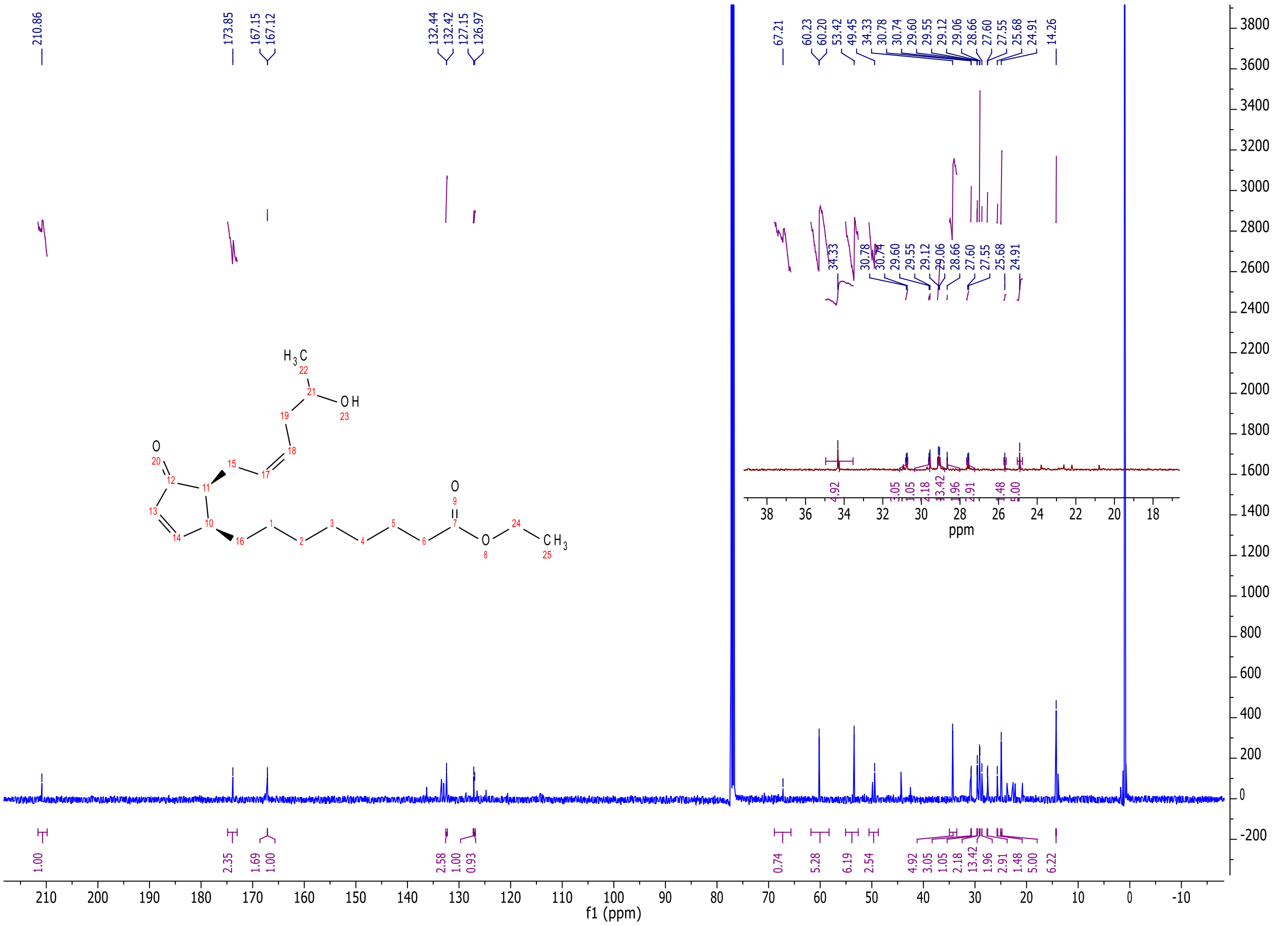
210.86
173.85
167.15
167.12
132.44
132.42
127.15
126.97
67.21
60.23
60.20
53.42
49.45
34.33
30.78
30.74
29.60
29.55
29.12
29.06
28.66
27.60
27.55
25.68
24.91
14.26
ppm
f1 (ppm)

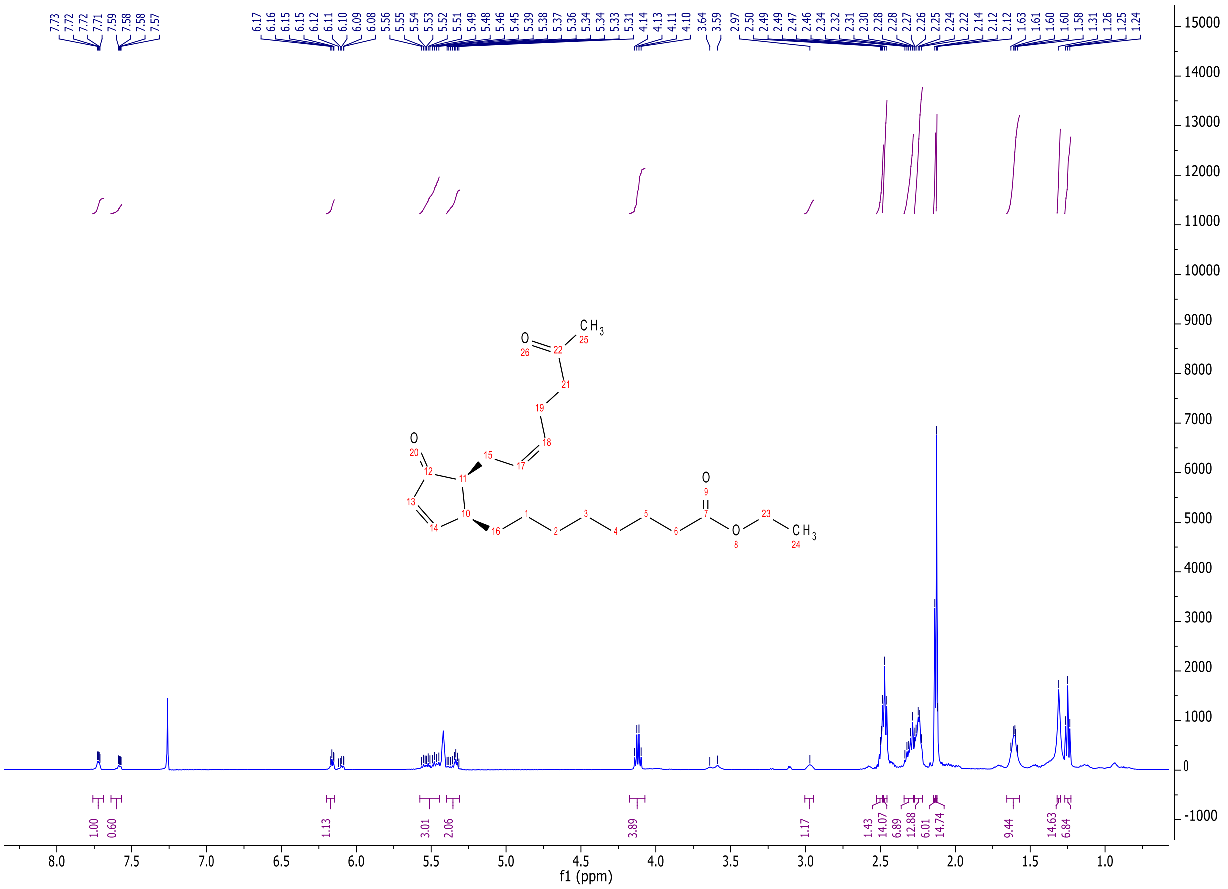
7.73 7.72 7.72 7.71 7.59 7.58 7.58 7.57
6.17 6.16 6.15 6.15 6.12 6.11 6.10 6.09 6.08 5.56 5.55 5.54 5.53 5.52 5.51 5.49 5.48 5.46 5.45 5.39 5.38 5.37 5.36 5.34 5.34 5.33 5.31 4.14 4.13 4.11 4.10 3.64 3.59 2.97 2.50 2.49 2.49 2.47 2.46 2.34 2.32 2.31 2.30 2.28 2.28 2.27 2.26 2.25 2.24 2.22 2.14 2.12 2.12 1.63 1.61 1.60 1.60 1.58 1.31 1.26 1.25 1.24
1.00 0.60 1.13 3.01 2.06 3.89 1.17 1.43 14.07 6.89 12.88 6.01 14.74 9.44 14.63 6.84
f1 (ppm)

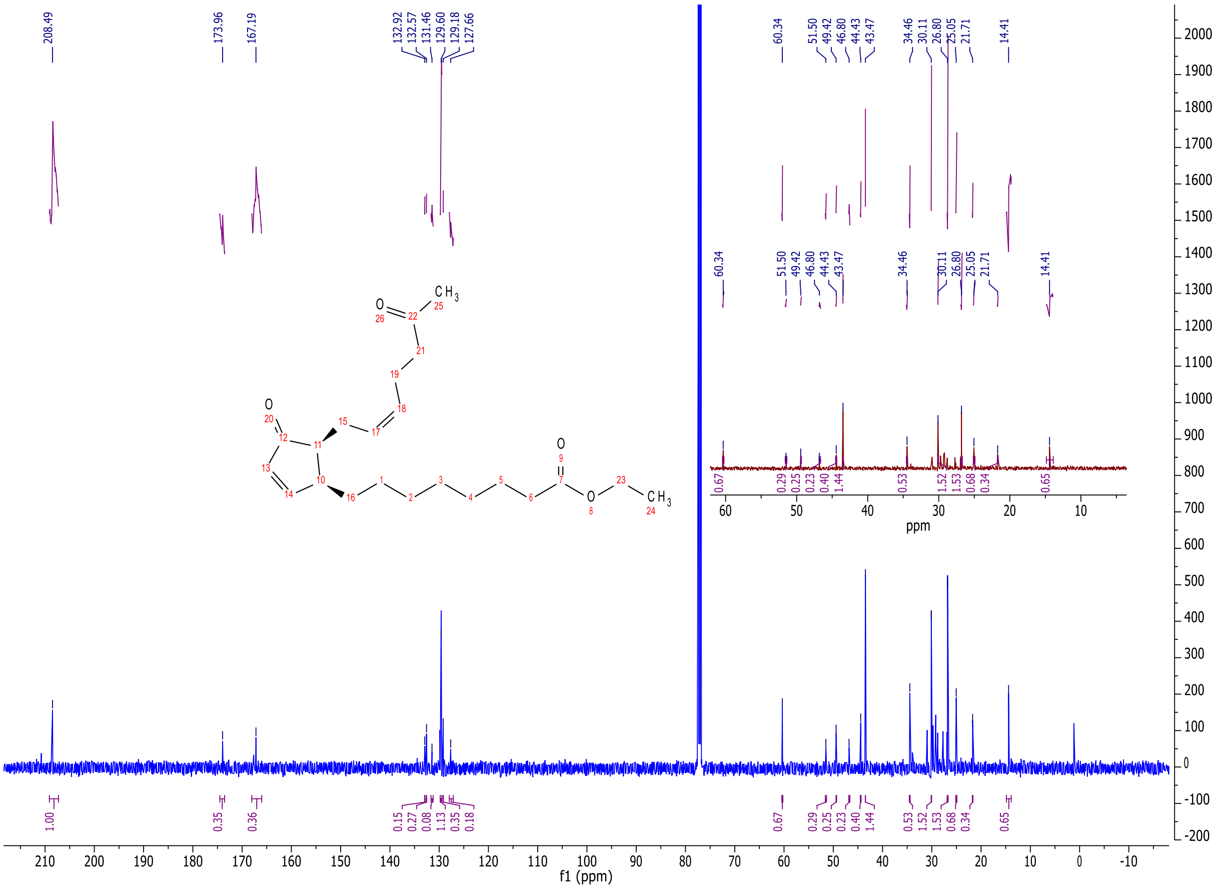
208.49
173.96
167.19
132.92 132.57 131.46 129.60 129.18 127.66
60.34
51.50 49.42 46.80 44.43 43.47
34.46 30.11 26.80 25.05 21.71
14.41
1.00 0.35 0.36 0.15 0.27 0.08 1.13 0.35 0.18 0.67 0.29 0.25 0.23 0.40 1.44 0.53 1.52 1.53 0.68 0.34 0.65
ppm
f1 (ppm)

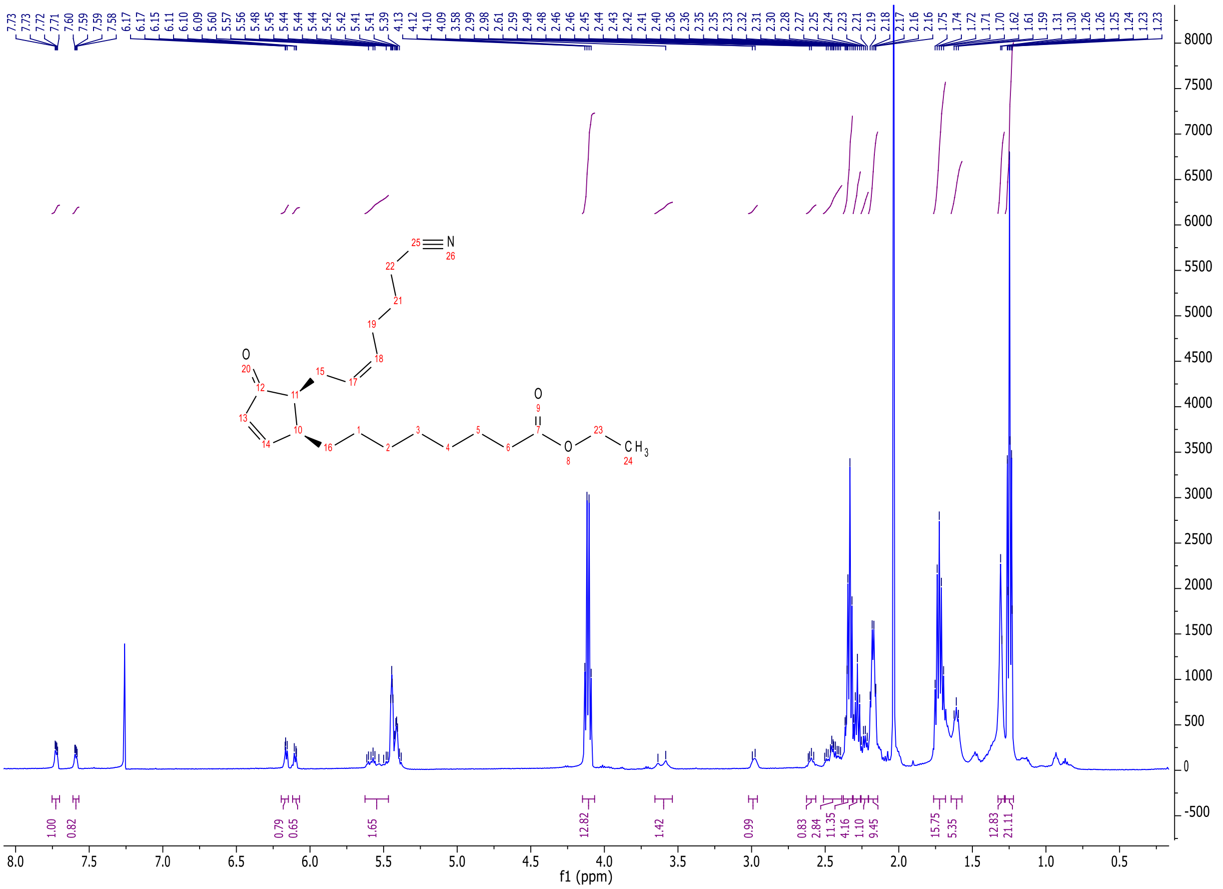

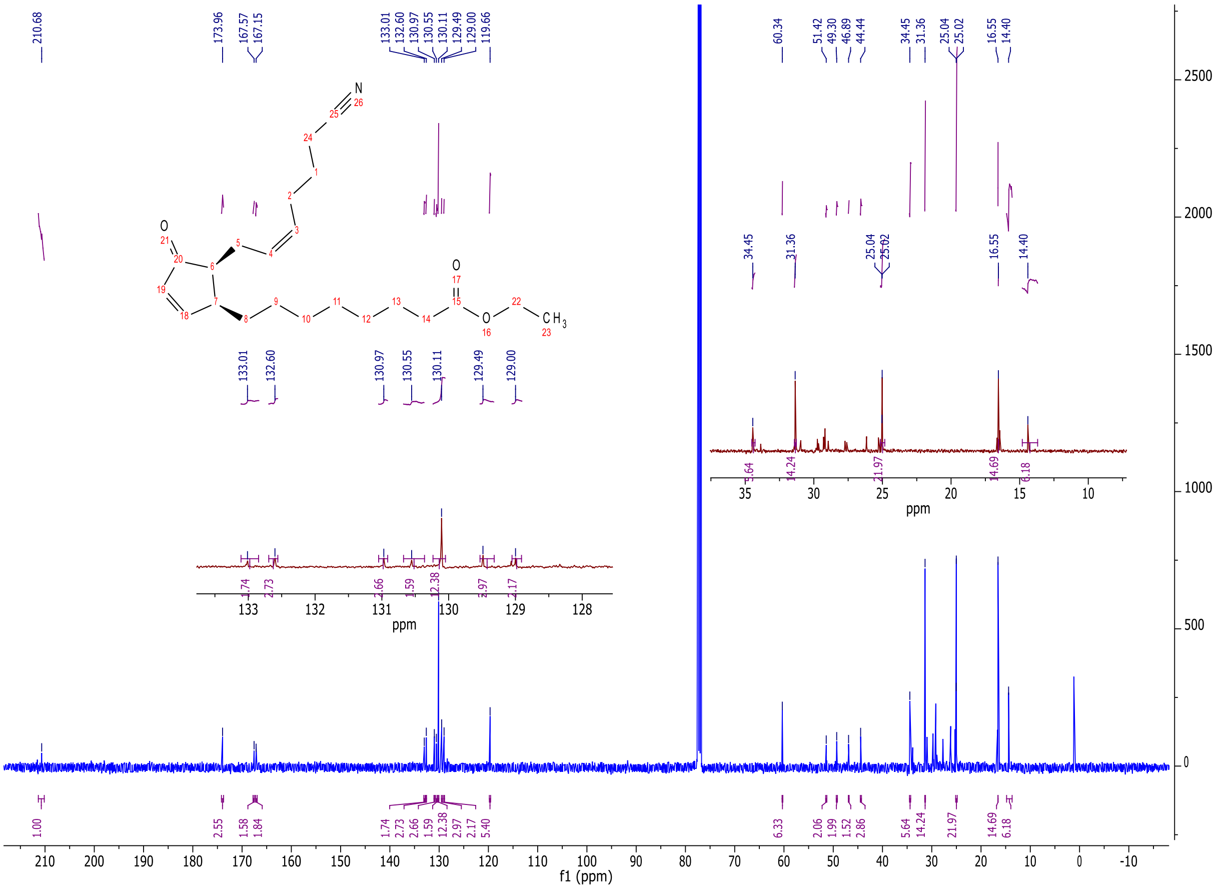

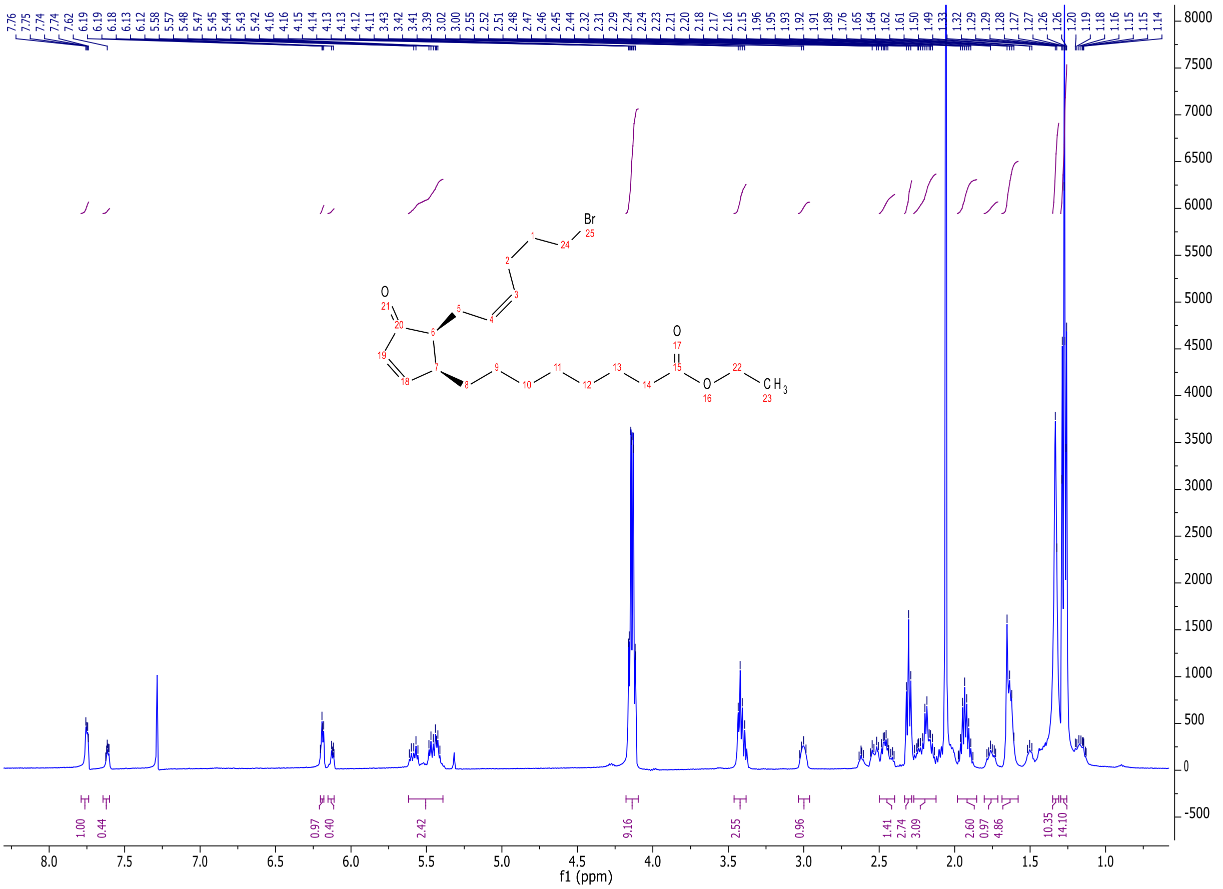

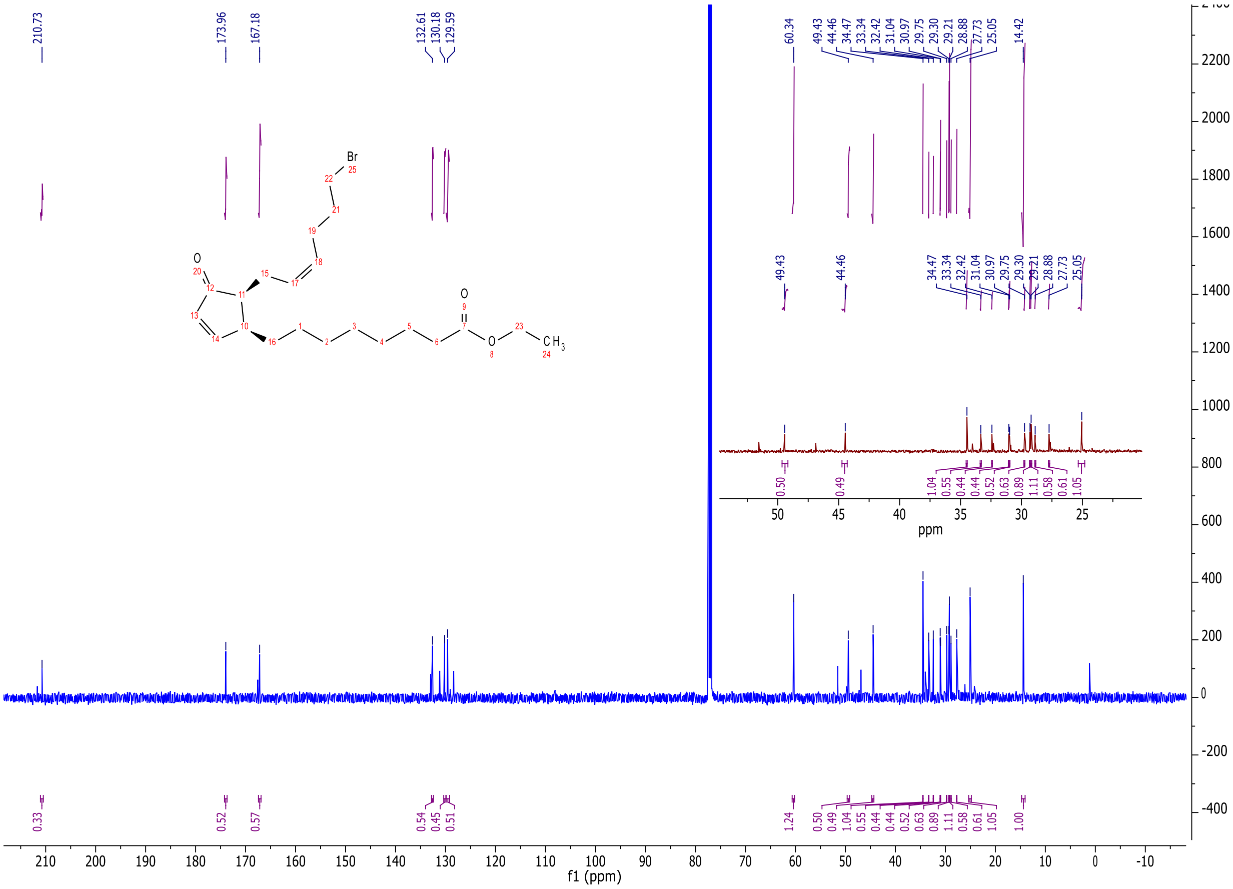

References

[1] I. Stenzel, B. Hause, O. Miersch, T. Kurz, H. Maucher, H. Weichert, J. Ziegler, I. Feussner, C. Wasternack, *Plant Mol. Biol.* **2003**, *51*, 895.

[2] M. Shizuaki, *Bull. Chem. Soc. Jpn*. **1984**, *57*, 3597.

[3] D. Maynard, S. M. Müller, M. Hahmeier, J. Löwe, I. Feussner, H. Gröger, A. Viehhauser, K. J. Dietz, *Bioorg. Med. Chem.* **2018**, *26*, 1356.

[4] S. W. Baertschi, C. D. Ingram, T. M. Harris, A. R. Brash, *Biochemistry* **1988**, *27*, 18.

[5] E. G. Bligh, W. J. Dyer, *Can. J. Biochem. Physiol.* **1959**, *37*, 911.

[6] P. A Grieco, N. Abood, *J. Org. Chem*. **1989**, *54*, 6008.
